# Combined Anti-Cancer Strategies Based on Anti-Checkpoint Inhibitor Antibodies

**DOI:** 10.3390/antib9020017

**Published:** 2020-05-20

**Authors:** Josée Golay, Alain E. Andrea

**Affiliations:** 1 Center of Cellular Therapy “G. Lanzani”, UOC Ematologia, Azienda Socio Sanitaria Territoriale Papa Giovanni XXIII, 24127 Bergamo, Italy; 2 Fondazione per la Ricerca Ospedale Maggiore, 24127 Bergamo, Italy; 3 Laboratoire de Biochimie et Thérapies Moléculaires, Faculté de Pharmacie, Université Saint Joseph de Beyrouth, Beirut 1100, Lebanon; alain.andrea@net.usj.edu.lb

**Keywords:** therapeutic antibodies, immune checkpoint inhibitors, cancer, microenvironment

## Abstract

Therapeutic monoclonal antibodies for the treatment of cancer came of age in 1997, with the approval of anti-CD20 Rituximab. Since then, a wide variety of antibodies have been developed with many different formats and mechanisms of action. Among these, antibodies blocking immune checkpoint inhibitors (ICI) have revolutionized the field, based on the novelty of their concept and their demonstrated efficacy in several types of cancer otherwise lacking effective immunotherapy approaches. ICI are expressed by tumor, stromal or immune cells infiltrating the tumor microenvironment, and negatively regulate anti-tumor immunity. Antibodies against the first discovered ICI, CTLA-4, PD-1 and PD-L1, have shown significant activity in phase III studies against melanoma and other solid cancers, alone or in combination with chemotherapy or radiotherapy. However, not all cancers and not all patients respond to these drugs. Therefore, novel antibodies targeting additional ICI are currently being developed. In addition, CTLA-4, PD-1 and PD-L1 blocking antibodies are being combined with each other or with other antibodies targeting novel ICI, immunostimulatory molecules, tumor antigens, angiogenic factors, complement receptors, or with T cell engaging bispecific antibodies (BsAb), with the aim of obtaining synergistic effects with minimal toxicity. In this review, we summarize the biological aspects behind such combinations and review some of the most important clinical data on ICI-specific antibodies.

## 1. Introduction

**Figure 1:**
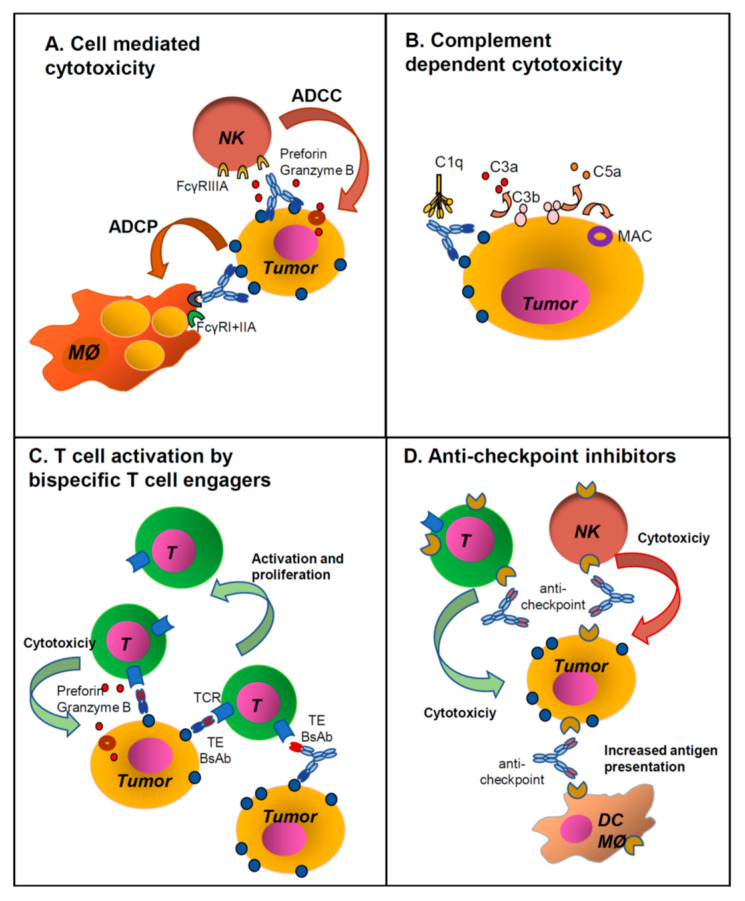
Major mechanisms of action of monoclonal and bispecific antibodies. Unconjugated IgG1 monoclonal antibodies (MAbs) work generally through activation of immune effector mechanisms through their Fc regions: (**A**) Antibody-dependent cellular cytotoxicity (ADCC) by NK cells and antibody-depndent phagocytosis (ADCP) by macrophages, (**B**) activation of the complement cascade. (**C**) T cell engaging bispecific antibodies (BsAbs) with or without Fc, act by binding a tumor antigen (TA) and CD3 on T cells (CD3 x TA). This induces activation of cytotoxic T cells which proliferate and kill the tumor cells. (**D**) Antibodies against immune checkpoint inhibitors (ICI) mostly block interaction of the ICI with their ligands, thus activating immune cells. This takes place via the Fab interaction with the ligand, blocking ICI function. In some cases, the MAbs may have a functional IgG1 Fc and eliminate ICI expressing cells through ADCC/ADCP or complement-dependent cytotoxicity (CDC). See also Table 1 for abbreviations.

Most anti-cancer monoclonal antibodies (MAbs) approved to date are unconjugated and have been shown to work, at least in part, through activation of innate immunity (macrophages, natural killer cells (NK) and complement) by the Fc region of the antibody (Figure 1A,B) [1,2,3]. Some bispecific antibodies (BsAbs), in contrast, activate adaptive immunity, in particular, T cells, through their anti-CD3 moiety, to kill tumor cells (Figure 1C) [4]. Antibodies against immune checkpoint inhibitors (ICI) have revolutionized the antibody field, being the first molecules to show significant activity even if not directed against a tumor antigen but against an immunomodulatory molecule. Indeed cancer cells have been known for many years to interact with immune cells present in the tumor [5,6,7]. Some of these immune cells have the potential of recognizing cancer cells and eliminating them but are often held in check by immune suppressor cells or signals rendering them tolerant or anergic. ICI are surface molecules expressed by tumor, stromal or immune cells that are involved in negatively regulating the anti-tumor immune response in the tumor microenvironment [7]. Thus, antibodies targeting ICI unleash anti-tumor immunity (Figure 1D). Equivalent to antibodies blocking ICI are the agonist, immunostimulatory antibodies that activate immunity by binding to the positive immune checkpoints and thereby triggering immunity against tumors in a relatively non-specific way [8]. In this review, we will discuss the biological rationale and clinical use of immunostimulatory and anti-immune checkpoint antibodies in combination with each other and with other therapeutic MAbs. Other biological molecules, such as cytokines and regulatory soluble proteins, also participate in the positive regulation of immunity in tumors and can also be used alone or conjugated to antibodies to shift the balance of immunity towards the control of tumor growth. Although of great interest, a discussion on the development and use of such therapeutic agents is beyond the scope of this review and will therefore not be discussed here. We refer the readers to some excellent recent reviews on the subject [9,10].

Antibodies directed against ICI, being independent from tumor antigens, have the advantage over anti-tumor antibodies of being potentially effective against a wide variety of tumors, including those which do not have adequate tumor antigens to be targeted specifically [11]. The disadvantage is, however, lack of specificity, resulting in the autoimmune side effects of these drugs. Furthermore, despite very impressive success in some types of cancer, ICI antibodies on their own are often not effective enough. Both the low efficacy in many tumor contexts and side effects have led to the development of alternative strategies, for example, targeting two or more ICI or an ICI together with other targets, using either BsAbs or combinations of MAbs [12]. This review examines the rationale as well as some of the pre-clinical and clinical data on these combined approaches. Parallel and complementary strategies also include combining ICI antibodies with standard chemotherapy radiotherapy and/or small targeted drugs, a theme too wide to be included here. Similarly, a discussion of side effects is beyond the scope of this work. We instead refer readers to recent reviews in the literature on these subjects [13,14,15]. Abbreviations used are summarized in Table 1.

## 2. Unconjugated MAb Structure and Function

Before examining the different options for combination strategies using ICI antibodies, we briefly summarize the structures of unconjugated MAbs, their functional properties and mechanisms of action according to antibody type and antigen target. Table 2 lists all the unconjugated MAbs approved by the US Federal Drug Administration (FDA) and/or European Medicines Agency (EMA) for cancer therapy up to May 1st 2020. In Table 2, the targeted antigens and formats of the MAbs are indicated. Isotype and format, at least in part, determine their function and are therefore important for understanding how two or more MAbs may cooperate with each other, as will be described below [16,17]. Most therapeutic anti-cancer MAbs targeting tumor antigens have an unmutated human IgG1 Fc (Table 2), the isotype best able to activate complement [3] (Figure 1B), and mediate antibody-dependent cellular cytotoxicity (ADCC) by NK cells, as well as antibody-dependent cellular phagocytosis (ADCP) by macrophages, i.e., effector functions of innate immunity [2] (Figure 1A). In some cases, modifications are introduced to enhance these activities, for example, glycoengineering of anti-CD20 obinutuzumab increases its binding to FcγRIIIA on NK cells and therefore enhances ADCC [1,2,16,17].

In contrast, the Fc of IgG2 and IgG4 isotypes are poor activators of innate immunity, because they bind poorly to the activating FcγRs, in particular FcγRIIA and FcγIIIA in man, and to the first component of the classical complement pathway, C1q. They therefore act mostly by inhibiting target functions through their Fab portion [18,19,20]. Of note is that wild-type human IgG4 has a unique Ser228 which allows for interchangeable disulfide bonds configuration and reshuffling of the 2 antibody arms in vivo, with the risk of generating hybrid IgG4 molecules derived from 2 different IgG4 molecules. The mutation of serine to proline at position 228 impedes this property, leading to more stable IgG4 in vivo. Therefore, all currently produced IgG4 therapeutic are mutated (S228P), to avoid Fab arm exchange [21] (Table 2).

Thus, activation of the immune system is the major mechanism of action of many anti-cancer IgG1 antibodies that target a tumor-specific antigen, such as those targeting CD20 (rituximab, ofatumumab and obinutuzumab), CD52 (alemtuzumab), CD38 (daratumumab), CCR4 (mogamulizumab), SLAMF7 (elotuzumab) and GD2 (dinutuxumab-β) (Table 2). Other anti-cancer MAbs that target receptors for growth or angiogenic factors expressed by tumor cells, like the epidermal growth factor receptors HER2 (trastuzumab and pertuzumab) and EGFR (cetuximab, panitumumab and necitumumab), or the vascular endothelial growth factor 2 (VEGFR2) (ramicirumab) act at least in part by inhibiting the growth or angiogenic signaling functions of the target receptors, and could therefore be constructed as IgG2 or IgG4 antibodies. Nonetheless, most of these have been designed as IgG1 and may therefore additionally induce innate immune activation for target cell elimination by the immune system (Table 2). Among these, only anti-EGFR panitumumab is IgG2.

With regard to ICI antibodies (Table 2), the major “scope” of these is to functionally inhibit the ICI by blocking its interaction with its ligand(s), for example, blocking programmed cell death protein 1 (PD-1) interaction with its ligands PD-L1 and PD-L2, thus inhibiting the negative signal normally induced through PD-1 [22]. This activity is mediated by the Fab region of the MAb (Figure 1D). Furthermore, the ICI may be expressed by immune cells which are not targeted to be eliminated (except in some cases, like Treg), but only to be activated to kill the tumor [22]. Thus, the Fc of ICI antibodies has generally been designed to be immunologically silent and most molecules approved so far for clinical use are IgG4, IgG2 or IgG1 with a mutated Fc which abolishes its interaction with FcγRs (Table 2) [23]. The only exceptions so far are avelumab (anti-PD-L1) and ipililumab (anti-Cytotoxic T-Lymphocyte-Associated protein 4, CTLA4).

In addition to immune effector activation, the Fc portion of all IgG isotypes interacts with FcRn, the neonatal receptor that recycles antibodies within cells, in particular endothelial cells of blood vessels, and rescues them from lysosomal degradation, thus prolonging their half-lives in vivo [24]. The half-life of IgG1, 2 and 4 is about 21 days. FcRn also promotes distribution of antibodies to some tissues by allowing transcytosis of antibodies across some epithelial barriers such as the gut [24,25].

## 3. Other Antibody Formats

Many unconjugated anti-tumor MAbs that have been tested in preclinical studies or early phase clinical studies have shown poor efficacy and/or toxicity, mainly due to low expression on tumor tissue or poor relative expression on tumor versus normal tissues [1]. This has led to the design of alternative formats, such as MAbs conjugated to radioactive payloads or to potent chemotherapeutic drugs (antibody-drug conjugates, ADC) and BsAbs [1,26,27]. The antibodies of these classes approved for cancer treatment are listed in Table 3. The mechanisms of action of these molecules are different from that of unconjugated MAbs. The major function of antibodies conjugated to radionuclides or drugs is to bring these cytotoxic agents very close to the tumor in order to promote direct killing. The Fc region in this case only has the function of facilitating purification and stabilizing the molecules in vivo and not that of activating innate immunity. 

BsAbs molecules are more versatile than MAbs, in that a considerable number of structural formats exist, with multiple combinations of two or more antigen specificities within one molecule [4,26,28]. The use of BsAbs offers the following advantages: (1) greater specificity (e.g., by combining two antigen targets, neither of which are fully tumor-specific), (2) increased or additional functional properties (e.g., neutralizing two different angiogenic factors, ICI or death receptors), and (3) new properties when the two targeted antigens display different functions (e.g., a tumor antigen and a receptor on effector cells such as T or NK cells [4,26,27]).

**Figure 2:**
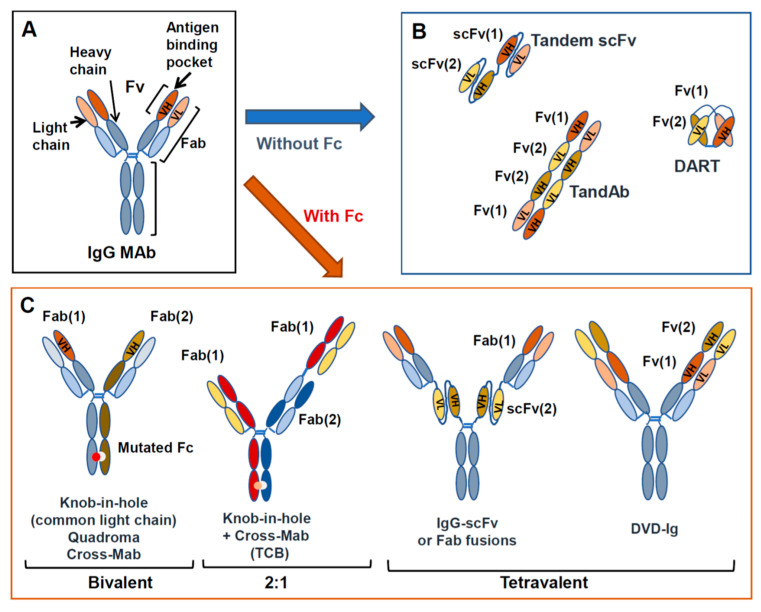
MAb and BsAb structures. Structure of a standard IgG (**A**) and examples of BsAbs, either lacking Fc (**B**) or containing an Fc region (**C**).

They can be bivalent (each arm being monovalent for each antigen) or tetravalent (with two binding sites for each specificity), or a mixture of the two, symmetric or asymmetric. More than 100 different formats have already been proposed and this number will no doubt increase in the future [28]. Examples of some of the different structures possible are shown in Figure 2B. The first group of BsAbs includes molecules based on the sole fragment variable (Fv) or fragment antigen binding (Fab) portions of two antibodies linked together, usually by peptide sequences, and lack Fc [26,28]. The second group of BsAbs carries a functional or mutated Fc linked to variable Fab or single chain fragment variable (scFv) domains carrying the two different specificities. Fc bearing BsAbs can be of different isotypes, wild-type or mutated, to enhance or diminish specific immune activating functions of Fc, according to the desired biological activity (see previous paragraph on MAbs). In some cases, the main function of Fc is to facilitate purification and stabilize the molecule in vivo [26,27,28].

Just as structures of BsAbs can be highly variable, so are their functions, which depend upon the antigen specificities that are targeted and the presence or absence of an immunologically active Fc [26]. Relatively few formats have reached the clinical stage and only three have been approved thus far. An important class of BsAb is the T cell engagers (TE), i.e., BsAbs that bind with one arm to the CD3 component of the T cells receptor and with the other arm bind to a tumor antigen [29]. These BsAbs activate T cells, after tumor target recognition, and bring T cells close to the tumor target so that the latter are efficiently killed (Figure 1C). Worth noting is that T cells belong to the adaptive component of immunity and thus, TE BsAbs activate immune cells by a different mechanism to those induced by Fc regions on MAbs (innate immunity, i.e., mostly NK cells and macrophages). Approved TE BsAbs include the mouse/rat chimeric Triomab catumaxomab (EpCAM × CD3), an orphan drug for Epidermal Cell Adhesion Molecule (EpCAM)-positive malignant ascites, and the tandem single chain variable fragment (scFv) bispecific T cell engaging (BiTE) antibody blinatumomab (CD3 × CD19) for refractory Philadelphia chromosome-negative acute lymphoblastic leukemia [30] (Table 3). It is expected that more bispecific or multispecific antibodies will be approved in the near future, including antibodies that engage immune cells other than T cells (e.g., NK, macrophages).

## 4. Immune Checkpoint Inhibitors

The development by William B. Coley of the first immune-based treatment for cancer, at the end of the nineteenth century, has highlighted immunotherapy as an important therapeutic modality to treat cancer [31].

In fact, the immune system is naturally strongly involved in cancer prevention, development and defense [32]. This “cancer immunosurveillance” hypothesis, initially postulated by Burnet and Thomas in the mid-20th century, originated from the evidence of the presence of tumor-associated antigens. This concept has developed into a wider and more complex “cancer immune-editing” version, described by Ikeda, Old and Schreiber, that encompasses three component phases, often referred to as the “3 Es”: Elimination, Equilibrium and Escape [33,34]. These three key events play a part in cancer elimination, dormancy and progression, respectively [35]. Tumor escape occurs when the immune system is unable to eradicate cancers that arise in the organism, driven by the accumulation of genetic alterations and epigenetic changes [36,37].

The more recently identified molecular mechanisms used by tumors to escape immune recognition can be classified into the following four categories: deficiencies in antigen processing and presentation [38], upregulation of inhibitory mechanisms and immunosuppressive function within the tumor microenvironment [39], deficiencies in costimulatory signals and thus in T-cell activation [40], and cancer cell immune resistance, also due, for example, to tumor aggressiveness [41]. Among escape mechanisms, recent promising advances in cancer immunology provide clear evidence in favor of the crucial role played by ICI in preventing tumor attack by the immune system [42]. In fact, ICI are important for the maintenance of self-tolerance, and normally turn off the immune response to prevent the body from destructing host tissues [43]. However, some cancers can exploit the immune checkpoint pathways by expressing ligands that can bind to these key immune regulators and thus attenuate the anti-tumor response and promote tumorigenesis [35,44]. The therapeutic antibodies, by blocking the inhibitory checkpoint proteins from binding to their corresponding antigens, prevent the “off” signal from being sent, ensuring the recognition of cancer cells by the host’s immune system and hence enhancing any preexistent anti-tumor immune activity that can lead to the elimination of malignant cells [45]. 

The Cytotoxic T-Lymphocyte Antigen 4 (CTLA-4) molecule, first characterized by Brunet et al. in the 1980s, was the first ICI to be targeted in a clinical trial in 2000, and ipilimumab, a CTLA-4 blocking antibody, was the first ICI antibody to be approved in 2011 by the FDA for the treatment of metastatic melanoma [46,47]. In 2014, the FDA approved another molecule, nivolumab, a PD-1 blocking antibody, as second-line treatment of unresectable or metastatic melanoma [48]. Drs. Tasuku Honjo and James Allison were jointly awarded the 2018 Nobel Prize for their contribution to the understanding of how the immune system is subject to inhibitory controls at PD-1 and CTLA-4 checkpoints. 

With regard to the mechanism of action, CTLA-4 is expressed on activated cytotoxic T-cells and Tregs. It binds to CD80 and CD86 expressed by antigen-presenting cells (APCs), competing on this function with the CD28 activating receptor, which is expressed by resting T cells but shows lower affinity for the CD80/86 ligand. Normally, an antigen-presenting cell will activate the T cell by binding both the T-cell receptor (TCR) and CD28. CTLA-4 acts as the “off” switch of this system by competing with CD28, triggering an inhibitory signal leading to a stop in proliferation. The blocking of CTLA-4 thus leads to the lifting of this “brake” and allows the T-cells to proliferate and become activated when they encounter a dendritic cell (DC) presenting a tumor antigen within a tissue [49]. CTLA-4 is also constitutively expressed by the FoxP3^+^CD4^+^CD25^+^ tumor infiltrating regulatory T cells (Tregs), which are important negative regulators of tumor immunity and of autoimmunity [50]. Thus, anti-CTLA-4 antibodies may inhibit CTLA-4 function in activated conventional T cells and may also downmodulate Tregs [23,50,51].

The second major checkpoint inhibitor PD-1 is expressed by activated T and B cells, NK and APCs. The two PD-1 ligands are PD-L1 (also called CD274 or B7-H1), expressed by numerous cells after exposure to inflammatory cytokines, and PD-L2, expressed predominantly by antigen-presenting cells. PD-1 inhibits T-cell function by preventing the activation of the signal induced by TCR activation via the SHP2 phosphatase that will dephosphorylate the activated proteins by the TCR pathway. In fact, in a tumor microenvironment, the expression of PD-L1 is enhanced by inflammation, thus allowing cancer cells to inhibit the cytotoxic anti-tumor response of T cells [52,53]. 

Following the considerable success of the previously discussed targets, several other immune checkpoint pathways have been discovered for the treatment of cancer. These are variably expressed by different immune cells, including T cells, NK, macrophages, Tregs, DCs, granulocytes, etc. Thus, a number of MAbs have been developed against these molecules and have been demonstrated, based on pre-clinical and some early clinical data, to have efficacy against different types of cancer. The most commonly targeted molecules are Lymphocyte Activation Gene 3 (LAG-3, CD223) [54], B and T lymphocyte attenuator (BTLA, CD272) [55], T-cell Immunoglobulin and Mucin domain-containing 3 (TIM-3, CD366) [56,57], V-domain Immunoglobulin Suppressor of T-cell Activation (VISTA) [58] and T cell Immunoreceptor with Immunoglobulin and ITIM domains (TIGIT) [59,60], the CD47-SIRPα receptor-ligand pair [61], as well as others. The major ICI and their ligands that are present targets of antibody development are listed in Table 4 (A). 

Similarly, anti-tumoral immunity may also be activated through the stimulation by agonist antibodies of activating rather than inhibitory checkpoints, including: CD27 [8], CD28 [8,62], Inducible T cell Co-stimulator (ICOS; CD278) [8,63], Glucocorticoid-Induced TNFR-Related protein (GITR) [8,64], NKG2D [65], OX40 [66] and 4-1BB (CD137) [67] (Table 4 (B)). Several MAbs and BsAbs are being developed against these molecules for clinical use.

## 5. Brief Overview of Clinical Data with Anti-Checkpoint Inhibitors as Monotherapy

Most ICI antibodies were first tested as monotherapies in a wide range of solid tumors and in some hematological cancers. Supplementary Table S1 summarizes the data of the most important phase II and III clinical trials with currently approved ICI antibodies. Only some salient aspects of these clinical studies will be discussed here, and we refer to Supplementary Table S1 and the original publications cited therein for more detailed results.

### 5.1. CTLA-4

As mentioned above, anti-CTLA-4 IgG2 tremelimumab was the first ICI antibody to be tested and gave some promising results, especially in terms of duration of response in melanoma patients [68,69]. Later, anti-CTLA-4 IgG1 ipililumab was also introduced in the clinic and this antibody showed significant activity alone or combined with vaccines or chemotherapy in Phase II and III clinical trials [47,70,71]. Later, studies established the 10 mg/kg q3w as the optimum tolerable dose and several Phase III studies firmly established the activity of ipililumab in melanoma patients. Simultaneously, ipililumab has been tested in Phase II or III studies in a number of advanced, recurrent or metastatic solid cancers, including ovarian, prostate and gastric cancers [47,72,73,74,75,76,77]. Clearly, in all these clinical contexts, combination strategies may offer an advantage over monotherapy and are being investigated. In particular, anti-CTLA-4 MAbs are being tested in combination with anti-PD-1 and PD-L1, and this will be discussed below.

### 5.2. PD-1

The anti-PD-1 IgG4 antibodies nivolumab and pembrolizumab were approved in 2014 for the treatment of advanced melanoma. In the following years, approval was extended to several other cancer types, including non-small cell lung cancer (NSCLC), head and neck, urothelial, renal and hepatocellular carcinomas and classical Hodgkin’s lymphoma (HL). In addition, nivolumab has been approved for the treatment of colorectal cancer (CRC) and small cell lung cancer (SCLC). Pembrolizumab has also been approved for gastric and esophageal carcinomas, Merkel cell carcinoma (MCC), cervical cancer and primary mediastinal B cell lymphoma (PMBCL). These approvals are based on clinical studies, some of which are summarized in Supplementary Table S1. Briefly, nivolumab showed a clear advantage in response over chemotherapy in advanced melanoma in Phase II and III clinical trials [78,79]. Also, in other cancers it has shown a good response and favorable safety profile [80,81,82,83,84,85,86,87,88,89,90,91,92,93,94]. In hematological tumors, nivolumab has shown a low response rate in follicular lymphoma (FL) [80] and diffuse large B cell lymphoma (DLBCL) [81], but higher efficacy in classical HL [82,83], which appears to correlate positively with 9p24.1 translocation and increased PD-L1 expression [94]. 

Interestingly, in some studies on adult T-cell leukemia/lymphoma (ATLL), nivolumab appears to increase leukemia progression (NCT02631746) [95,96], indicating that in this disease, PD-1 plays a tumor-suppressive role. 

Pembrolizumab has similarly shown efficacy in terms of overall response rate (ORR) and survival (OS) in several clinical contexts [97,98,99,100,101,102,103,104,105,106,107]. Notably, in melanoma, pembrolizumab showed a significantly better ORR with a favorable toxicity profile compared to ipililumab [75]. The antibody induced better OS compared to chemotherapy in NSCLC and urothelial cancers, always with a favorable toxicity profile [106].

So far, pembrolizumab and nivolumab appear therefore to have similar activity. Interestingly, their standard dosing is different, nivolumab being generally given at 3 mg/kg every 2 weeks, whereas a 10 mg/kg, or more recently, a flat 200 mg, dose of pembrolizumab is being administered every 3 weeks. 

A new anti-PD-1 (cemiplimab) has been approved more recently (2018) for the treatment of squamous cell carcinoma on the basis of Phase II studies [108].

### 5.3. PD-L1

PD-L1 is expressed in several cancer tissues, including MCC, melanoma, hepatocellular carcinoma (HCC), lung cancer, breast cancer, lymphoma and myeloma, as well as others. It is expressed by cancer cells as well as by some immune cells within the tumor microenvironment [109].

Three anti-PD-L1 MAbs have been approved so far for anti-cancer treatment, one IgG1 (avelumab, Fc competent) and 2 IgG1 mutated (atezolizumab and durvalumab, Fc silent) (Table 2) [110,111,112,113]. Out of all ICIs, avelumab is presently approved by the FDA for the treatment of metastatic MCC, urothelial carcinoma (UC) and renal cell carcinoma (RCC) [114]. Indeed, it has shown promising clinical activity in these diseases (Supplementary Table S1) [115,116,117,118]. Although it has not demonstrated superior therapeutic activity compared to chemotherapy in NSCLC, its safety profile was favorable [118]. In contrast, efficacy appears low in advanced gastric cancers [115]. Like other ICI antibodies, it is now also tested in combination with chemotherapy in solid cancers.

Atezolizumab is one of the first approved anti-PD-L1 for the treatment of UC and approval was extended subsequently for lung cancers, bladder carcinoma and triple-negative breast carcinoma, where it has shown efficacy and a favorable toxicity profile [119,120,121,122,123,124]. It is being further tested in combination with chemotherapy or antibodies such as bevacizumab, as well as with anti-CTLA-4 MAbs (see below).

Durvalumab is another anti-PD-L1 approved in 2017 for the treatment of advanced bladder cancer and subsequently for unresectable stage III NSCLC (Supplementary Table S1) [125,126]. More recently, it is being tested with or without tremelimumab in a number of clinical conditions (see Section 7 below and Table 5).

In conclusion, ICI antibodies directed against CTLA-4 or PD-1 and PD-L1 have shown significant activity in several solid cancers, most notably, melanoma and NSCLC and in some hematological neoplasms, in particular classical HL. Nonetheless, in most cases, response to monotherapy is insufficient. Furthermore, much effort must be invested into defining biological markers that may correlate with response and/or toxicity. Indeed, many trials have asked the question whether PD-L1 or PD-1 expression as well as other markers could be predictors of response, with mixed results [98,104]. Indeed, it is likely that additional factors also determine response, such as tumor antigenicity, poor tumor immune infiltration, the presence of several immune inhibitory mechanisms and pathways. Clearly, identifying reliable biomarkers to predict response is currently one of the most important challenges.

Finally, many antibodies against the same or novel ICI are in development and some have already entered clinical trials, alone or in combination with other drugs, as further discussed below. Reviews have been published on these novel ICI and results from efficacy studies are eagerly awaited [42,127].

## 6. The Possible Role of Antibody Isotypes in the Efficacy of ICI Antibodies

As already stated above in Section 5, many ICI antibodies have been produced in an IgG2, IgG4 or Fc silent IgG1 format. This diminishes their ability to bind to FcγRs on NK, B and myeloid cells, and thus considerably reduces their ability to activate these cells and also reduces their potential to activate complement. This is because the major focused action of the ICI antibodies is to activate immunity through inhibition of ICI. Indeed, Fc-mediated killing of immune target cells such as T cells expressing ICI is often unwanted. Nonetheless, the elimination of some immune cells that express ICI, for example, Treg or other suppressor cells, may also be useful in some circumstances and in these cases, an active IgG1 Fc may be useful for efficacy. Therefore, some pre-clinical studies have attempted to define the effect of using different IgG isotypes. These studies are nicely reviewed by Chen et al. [23]. Briefly, the available data in animal models suggest that for some ICI, an IgG1 isotype capable of binding Fcγ (with a so-called competent Fc) induces a better response than isotypes with weak FcγR binding capability (bearing a so-called silent Fc). Thus, antibodies targeting PD-L1, CTLA-4, TIGIT, VISTA and B7-H3 work most effectively if they carry a competent Fc. Instead, antibodies against PD-1, TIM-3, LAG-3 and CD73 have maximum efficacy when constructed with a silent Fc [23]. It is important to note that these conclusions are based on only limited preclinical data [51], mostly in mice, and may not be representative of man and of all tumor contexts. There is little doubt however that a better understanding of the role of Fc in the clinical activity and toxicity of different ICI antibodies would be of great value. 

Perhaps the most studied example of the effect of isotype on efficacy is that the anti-CTLA-4 IgG1 isotype may be preferred over IgG4 or IgG2, because one possible mechanism of anti-CTLA-4 antibody activity is the killing of Tregs [23,51,128]. Evidence in favor of IgG1 with regard to anti-CTLA-4 is that: (1) a correlation between efficacy and Treg killing has been observed in animal models, (2) antibodies that do not block binding of CTLA-4 to its ligand CD80/86 are nonetheless active in these models and (3) preliminary reports from mouse and macaque studies suggest that a defucosylated variant of ipililumab may be more effective [23,128]. However, in humans, available data have not confirmed that Treg depletion correlates with clinical response. Indeed, evidence suggests that neither ipililumab nor tremelilumab deplete Treg in melanoma and other solid tumor tissues in man and that both are equally able to expand CD4 and CD8 cells [129]. A direct comparison of ipililumab and tremelilumab at equal doses in clinical trials would be the only clear-cut approach to define whether there is any difference on response of patients to IgG2 tremelilumab and IgG1 ipililumab (Supplementary Table S1). 

PD-L1 is an ICI also frequently expressed by tumor cells themselves and, thus, actual elimination of PD-L1-positive cells by IgG1 antibodies may also be a useful property of these antibodies. There are indeed some preclinical data suggesting that engagement of FcγRs may enhance efficacy of anti-PD-L1 antibodies. But this question remains to be resolved; for example, there is the possibility that such antibodies may deplete PD-L1-positive APCs and other useful immune cells [23]. As already mentioned, atezolizumab and durvalumab have mutated IgG1 CH2 domains (Fc silent), whereas avelumab bears an unmutated functional IgG1 Fc. Unfortunately, clinical studies obtained so far do not allow to resolve the question as to whether the wild-type or mutated IgG1 format is more favorable for anti-PD-L1 activity (Supplementary Table S1).

Thus, although the fact that MAb isotype may affect the efficacy or mechanism of response to ICI antibodies, and the isotype choice has to be taken into consideration when designing an antibody, still much work is required to better define the role of Fc in the clinic for both old and new ICI antibodies. The possible effect of Fc on toxicity also needs to be evaluated. 

## 7. Combination of ICI Antibodies and Other MAbs or BsAbs

As stated above, ICI antibodies have shown significant and even impressive clinical activities against some cancers, but not against others, and generally, the impressive clinical activity is manifest only in a subset of patients. Indeed, tumors are known to develop multiple mechanisms for evading immune surveillance, and these as well as their high proliferation potential, apoptosis resistance, enhanced migratory potentials and angiogenetic properties are all factors that may contribute to resistance or relapse from monotherapy with ICI antibodies [130]. Thus, combined strategies are likely to be more effective, either by retargeting two different ICI or by targeting an ICI together with a different mechanism of tumor escape/aggressiveness or with a different expression level in the specific tumor. Antibodies are good candidates for such combined strategies, either as a combination of two MAbs or in the form of BsAbs, and many different molecules are already in the clinic or in development for such approach. Before describing the clinical results and programs for these specific combination therapies, we will briefly review the rationale and supporting preclinical data. 

### 7.1. Rationale for Combining Anti-CTLA-4 with Anti-PD-1/PD-L1 

The combination of antibodies targeting two different ICI has its rationale when the clinical results with single agents have not shown sufficient therapeutic activity. One mechanism of resistance to ICI antibodies is considered to be the low immunogenicity of some tumors and/or low infiltration of the tumor tissue by immune cells. There can also be resistance due to the presence of alternative pathways of immunosuppression, not targeted by the ICI antibody used, as well as low expression of the targeted ICI [37]. Thus, it was expected that targeting two different pathways which are active in different phases of immune activation or in different immune cell types may enhance and perhaps lead to a synergistic effect. 

In particular, targeting CTLA-4 and PD-1/PD-L1 is expected to act on both the induction phase and effector phase of T cell-mediated immunity [32]. Indeed, CTLA-4 is expressed by resting T cells and is thought to compete with the CD28 second activation signal of T cells, for B7-1 (CD80) and B7-2 (CD86) on APC family protein binding (CD80 and CD86). CTLA-4 inhibits TCR phosphorylation. In contrast, PD-1 is induced after T cell activation and is thought to play a role in the downmodulation of the T cell response and in tolerance. It was therefore reasonable to expect that the CTL4 and PD-1 pathways may complement each other and that antibodies targeting both these molecules would be synergistic in vivo. Thus, many clinical trials have been initiated combining these agents and the clinical results obtained so far are summarized below. 

### 7.2. Clinical Results Obtained with Anti-CTLA-4 and Anti-PD-1/PD-L1 Combinations

A number of efficacy studies have been conducted to evaluate the combination of anti-PD-1/PD-L1 with anti-CTLA4 antibodies. These are listed in Table 5. In particular, combination of anti-PD-1 nivolumab and anti-CTLA-4 ipililumab has been tested in Phase II or III studies in a number of solid cancers, from melanoma [131,132,133,134], to renal [135,136], colon [137,138], esophageal [139], sarcoma [140], lung cancers [141,142,143,144] and mesothelioma [145] (Table 5). One of the principal motivations behind many of these trials were the early results combining these MAbs in the context of advanced melanoma, where Phase II and III studies clearly demonstrated the greater efficacy of the antibody combination over monotherapy (and also showing the better efficacy of anti-PD-1 over anti-CTLA-4 monotherapies). Indeed ORR, OS and progression-free survival (PFS) were all consistently and significantly increased in the patients treated with the combined antibodies and the results of Phase II and III studies were in line with each other [131,132,133,134] (summarized in Table 5). 

The combination has also been tested in Phase III studies versus chemotherapy in RCC and NSCLC. Also, in these cancers, the combination resulted in better ORR and OS, although the PFS improvement was nil or modest [135,136,141,142]. Nivolumab and ipililumab have also been tested in Phase II studies in sarcoma (NCT02500797) and CRC (NCT02060188), with some improvement in response (ORR, OS and PFS) with respect to nivolumab monotherapy [137,138,140]. These data will need to be reinforced in larger Phase III studies. In contrast, in several contexts (esophagogastric carcinoma, SCLC and pleural mesothelioma), the combination of nivolumab with ipililumab in Phase II trials has failed to show a significant advantage over nivolumab monotherapy, especially considering OS and PFS [139,143,145] (Table 5).

Results of several clinical efficacy studies combining anti-PD-L1 durvalumab with anti-CTLA-4 tremelimumab have also been reported. In squamous cell carcinoma of the head and neck, the combination strategy has not improved ORR, PFS and OS over one MAb alone in a Phase II study [146,147] (Table 5). Moreover, a large Phase III study indicates that the combination does not improve response over durvalumab alone and neither over chemotherapy [148]. Similarly, the combination appears to have very limited efficacy in metastatic pancreatic duct adenocarcinoma [149]. Finally, a large Phase III study of durvalumab with or without tremelilumab compared to chemotherapy as a first-line treatment of NSCLC, enrolled 1118 patients. Subgroup analysis of 488 patients with >25% PD-L1 expression showed an improved overall survival in the durvalumab ± tremelilumab group compared to chemotherapy. Addition of tremelimumab to durvalumab did not appear to increase responses, but it did lead to more side effects which resulted in treatment discontinuation [150]. This study suggests that selection of patients with higher ICI expression in the tumor may be appropriate for clinical studies, even though a correlation between ICI and response has not always been observed [142,150].

Altogether, the results obtained so far suggest that, as for monotherapy, not all tumor types respond to ICI antibodies, either alone or in combination. Nonetheless, some tumor types have shown significantly improved results. Improved response is unfortunately often accompanied by increased toxicity, which in some cases explains the lack of effect of combination on overall survival [139,143].

### 7.3. Combinations Using Novel ICI Antibodies

Some correlation was observed between expression of PD-L1 or PD-1 within the tumor microenvironment and response to antibodies targeting this pathway. This suggested that in cases showing low expression, other immune inhibitory pathways may be more important, leading to the design of antibodies targeting ICI other than CTLA-4 and PD-1/PD-L1, that are either overexpressed in specific types of tumors and/or involved in downmodulating anti-cancer immunity mediated by T cells, NK and myeloid cells [40,151]. Table 6 lists all the combination of antibodies, targeting two or more ICI or immune stimulators, being tested in the clinic at the present time. A wide variety of combinations are being tested against many different cancers. Most often, and for obvious reasons, the first antibody is an approved anti-PD-1/PD-L1 or anti-CTLA-4, combined with more novel ICI antibodies.

One frequently used targeted molecule in these combinations is LAG-3, which is a CD4-like molecule expressed by tumor infiltrating lymphocytes (TILs), by activated CD4 and CD8 T cells, Tregs, B cells and DCs. It interacts with MHC class II and inhibits CD4 T cell proliferation, cytokine release and CD8 effector functions. It is overexpressed in some tumors and its combination with classical ICI antibodies is expected to improve responses. Thus, many Phase I/II trials and one Phase II/III trial have been started with anti-LAG-3 combined with the more established ICI antibodies. Results from these studies are awaited [152]. 

TIM-3 is another molecule of interest. It is expressed by exhausted T cells, Tregs, B cells, NK cells, DCs, macrophages and mast cells and binds to galectin 9, High Mobility Group Box 1 (HMGB1) and Carcinoembryonic Antigen-Related Cell Adhesion Molecule 1 (CEACAM-1). It is overexpressed by some tumors. The mechanism of inhibition of T cell proliferation and TH1 responses by TIM-3 are still unclear and the subject of controversy [152]. Nonetheless, the frequent co-expression of TIM-3 with PD-1 on exhausted T cells and TILs has offered a rationale for combined use of anti-PD-1 and anti-TIM-3. Moreover, experiments in some mouse models have provided evidence for the efficacy of this combination [153]. Thus, several mostly Phase I studies of anti-PD-1 and anti-TIM-3 have been initiated in advanced and metastatic solid tumors, but the data are not yet available. 

TIGIT is another ICI that has gained much interest and is being tested in combination with “classical” ICI antibodies. It belongs to the family of nectin and nectin-like proteins and is expressed by NK and T cells, including CD4, CD8 and Tregs [154]. It binds with high affinity to CD155 (Polio Virus Receptor, PVR), to CD112, but with lower affinity to the CD113. It competes with DNAM-1, an activating receptor, for CD155 binding and may inhibit T and NK cell activation in this way, similar to competition between CTLA-4 and CD28 binding to B7 molecules. In addition, TIGIT interaction with CD155 induces tolerogenic signals, such as IL-10 production and decreased secretion of IFN-γ and IL-12 by DCs, resulting in decreased priming and proliferation of T cells, and induction of a M2-type phenotype in macrophages. These data, and the fact that TIGIT is overexpressed in the microenvironment of several tumor types, such as multiple myeloma, melanoma, gastric cancers and AML, has led to the development of several anti-TIGIT antibodies, all IgG1 with either Fc competent or silent Fcs, and the initiation of Phase I/II clinical studies alone or in combination with PD-1/PD-L1 blockade [154].

PVRIG is related to TIGIT and binds to CD112 but not to CD155. It inhibits T cell cytotoxicity and PVRIG inhibition increases anti-tumor response [155]. Furthermore, PVRIG blockade appears to cooperate with anti-PD-1 [156]. A phase I clinical trial has been started combining antibodies targeting PVRIG and PD-1 in advanced solid tumors (NCT03667716). 

Other combinations aim to more specifically target cell types other than T cells or Tregs, in particular, NK cells (NKG2A) [157]. NKG2A is a well-known inhibitory receptor of NK cells. Preclinical studies suggest that its use in combination with the anti-PD-1/PD-L1 pathway inhibitors may reinforce NK cell activation [158]. This combination is being tested in clinical studies (NCT03833440).

Other molecules being actively investigated and tested as ICI belong to quite different pathways of immune inhibition. This is the case of CD73 which is part of the complex of cell surface enzymes, regulating adenosine metabolism, which includes CD39 and adenosine receptors. CD39 is an ATPase which hydrolizes ATP and ADP to AMP. CD73 is an ecto-5′-nucleotidase which hydrolizes, in turn, AMP to adenosine. The adenosine receptors A2a (ADORA2A) and A2b (ADORA2B) in turn increase cAMP levels in response to ligand and downmodulate the immune response mediated by T cell, NK and macrophages. All these enzymes therefore cooperate to downmodulate inflammation and the immune system in physiological conditions. They are expressed by many cell types, including tumor cells, stromal cells, immune cells and are upregulated on Treg [159]. Thus, blocking CD73 reduces adenosine production and activates immune cells. Several anti-CD73 MAbs have been introduced in the clinic, all bearing a silent Fc, because CD73 is ubiquitously expressed and the aim of the antibodies is to block CD73 function and not eliminate the target cells (see above). Indeed, evidence suggests that a major mechanism of some anti-CD73 MAbs, like BMS-986179, is to internalize CD73 leading to decreased cAMP production. Many Phase I/II clinical trials have also been started combining anti-CD73 and anti-PD-1/PD-L1 MAbs against hematological tumors. Anti-adenosine receptors A2a and A2b antibodies are also being developed [160].

CD47 is a ubiquitous protein whose physiological function is to send a “*do not eat me*” signal to macrophages and inhibit phagocytosis of healthy cells. The CD47 ligand is CD172a (SIRP1α) expressed by macrophages, including Kupffer cells in the liver, granulocytes, DCs and neuronal cells. SIRPα bears a negative regulatory domain (ITIM) in its cytoplasmic tail so that interaction with CD47 inhibits positive signal triggering for activation of SIRPα-positive cells and phagocytosis, for example, by macrophages. Thus, healthy cells that express CD47, in particular erythrocytes, send a negative signal to macrophages and blocks their phagocytosis, whereas damaged or old cells (aging erythrocytes as well as others) have lower levels of CD47 and are rapidly eliminated by macrophages. Increased CD47 has been shown in some tumors, like AML, CML, B-NHL and numerous solid tumors, as well as in cancer stem cells, thus providing a mechanism for immune evasion [161]. Blocking CD47/SIRPα increases phagocytosis by macrophages and antigen presentation by DCs, thus promoting adaptive immunity. In addition, anti-CD47 may induce cell death of tumors cells expressing this molecule, but this possible mechanism is uncertain [162]. Blocking CD47/SIRPα interaction has been shown to synergize in vitro and in vivo with some therapeutic MAbs, like rituximab, which act at least in part by inducing phagocytosis of CD20-positive tumor cells [61]. Anti-CD47 may also enhance the efficacy of other standard therapeutic MAbs, such as cetuximab and trastuzumab [163]. Finally, pre-clinical models suggest a synergy between anti-CD47 and anti-EGFR/HER2 antibodies of IgA isotype, through the action of neutrophils [164]. The use of an anti-CD47 IgG4 (Hu5F9 G4) is therefore entering the clinic, alone or in combination with these MAbs (Table 6). In this case, the silent Fc version of anti-CD47 is preferred to mediate the blocking of the CD47–SIRPα interaction rather than to promote Fc-mediated activities. 

In addition to what has been said about possible synergies, it is important to note that combining two different ICI antibodies bears with it the intrinsic risk of increased toxicity, in particular, enhanced autoimmune and allergic reactions. This has indeed been shown to be the case in a number of clinical studies [165].

### 7.4. Combining ICI and Immune Stimulating Antibodies

A corollary to inhibiting two or more ICI is to combine them with immune activators. Well-known immune stimulators include CD137, GITR, OX40 and CD27, as well as others, most of which belong to the TNFR superfamily. They are expressed by T cells, in some cases also Treg (GITR, OX40) and variably by other immune cells types. As for inhibitory molecules, the list of these agents as potential targets for cancer immunotherapy is growing and a more precise description of these molecules is beyond the scope of this review. We instead refer the readers to several recent articles [166,167]. Several agonistic antibodies have been developed against the immune activators. After ligand binding, these antibodies induce activating pathways in the targeted cells (e.g., via MAPK and NFkB signaling), most often T cells or antigen-presenting cells which may be present within the tumor microenvironment [167]. Some antibodies like anti-GITR and OX40 may also deplete Tregs [64,168]. 

Thus, immune-stimulating antibodies have shown anti-tumor activity in preclinical models and are therefore being tested in Phase I or Phase I/II clinical studies, either alone or in combination with standard MAbs (in particular rituximab and cetuximab), or with ICI antibodies, mostly anti-PD-1/PD-L1 or CTLA-4 in various solid and hematological tumors [169]. The combinations of ICI and immune stimulatory antibodies that have presently entered clinical trials are listed in Table 6. As noted above, enhanced autoimmune and allergic reactions are also likely to occur as a consequence of using a combination of ICI and immune-activating antibodies. 

### 7.5. Combination of an ICI Antibody with a Standard Anti-Tumor MAb

One interesting combination strategy is to use an ICI antibody with standard Fc competent IgG1 anti-tumor antibodies, like rituximab, cetuximab, trastuzumab or daratumumab. Indeed, these latter antibodies are known to activate innate immunity: ADCP, ADCC, as well as complement [2,3]. This results in the release of pro-inflammatory cytokines and chemokines, including complement anaphylatoxins C3a and C5a [170]. These may favor the recall of immune cells to the tumor, facilitating further immune control of tumors [171]. Thus, combining anti-tumor MAbs with ICI antibodies that activate T cells or stimulate hyperactivate innate immune cells may lead to synergistic effects. 

Interestingly, some MAbs, like trastuzumab and cetuximab, have also been shown to upregulate the expression of ICI, like PD-1, PD-L1 or TIM3 [172,173]. Also, EGF stimulates PD-L1 expression [174,175]. Although the interplay between EGFR signaling and the PD-L1 /PD-1 pathway is complex and still needs to be fully understood [176], these observations offer a rationale for using anti-EFGR cetuximab in combination with anti-PD-1 or PD-L1 in HNSCC or NSCLC (Table 6). Indeed, several clinical studies combining cetuximab or trastuzumab with anti-PD-1/PD-L1 have been initiated [177]. Results from one Phase II study combining pembrolizumab with trastuzumab for trastuzumab-resistant advanced breast cancer has been reported (NCT02129556). The data show promising clinical activity of the combination in the PD-L1-positive subgroup (Table 4) [98]. 

In other cases, high ICI expression has been detected in tumors known to be responsive to a specific MAb. For example, PD-1 and PD-L1 have been shown to be expressed in some B-Non Hodgkin’s Lymphoma (B-NHL) subtypes and to correlate with poor prognosis [178,179]. Thus, the combination of anti-PD-1 and anti-PD-L1 with anti-CD20 antibodies effective in B-NHL is quite an obvious choice, which is being tested in the clinic. 

Standard MAbs may also be combined with agonist immune-activating antibodies in a strategy to increase immunity against tumor, the first MAb directing the innate immune system specifically to the tumor and the other offering a boost to the same or other players of the immune system [180].

### 7.6. Combination of an ICI Antibody with a T Cell Engager Antibody

Another combination approach is to use an ICI antibody with TE BsAbs (Table 6). Several such combinations are already being tested in the clinic, in particular using the approved BsAb (BiTE) CD3xCD19 blinatumomab [181]. The biological rationale is, also in this case, to reinforce, through the ICI antibody, an immune cell activation that is already induced by the TE antibody. More specifically, the TE bispecific activates T cells, induces their proliferation and the killing of the tumor cells by CD8^+^ cytotoxic T cells. Increased PD-1 and PD-L1 expression on a tumor or in its microenvironment has been observed following blinatumomab administration and this has been suggested as a mechanism of resistance to this BsAb [182]. Thus, an obvious strategy is to combine blinatumomab with anti-PD-1 or PD-L1 antibodies to control these resistance mechanisms, and several such trials are being carried out. 

Other data suggest that the presence of Tregs inhibits the response to blinatumomab, so combination with antibodies that may deplete Tregs, like anti-CTLA-4, is another logical approach that is being pursued [181]. The paradigm may not only reinforce the T cell-mediated anti-tumor response already activated by the TE BsAb, but it may also activate other immune cells that would not be normally and specifically involved in the response, with potentially additive or synergistic effects. Combinations of ICI antibodies with TE BsAbs other than blinatumomab are also being tested, including the CD20 × CD3 IgG4 BsAb, REGN1979, for B-NHL, and the gpA33 × CD3 DART-type BsAb, MGD007, for colon cancer (Table 6). The rationale behind these combinations is similar to that for blinatumomab, since all these TE antibodies have been designed to activate T cells through TCR and have no Fc or a silent Fc. As in other cases, the drug combinations may lead to higher efficacy but also increased side effects, which will need to be carefully evaluated.

### 7.7. Combination of Antibodies Targeting ICI and ReceptorActivator of Nuclear Factor kB Ligand (RANKL) Antibodies

The mechanisms by which RANK/RANK Ligand (RANKL) may interact with ICI has been nicely reviewed by van Dam [183]. RANKL is a transmembrane and soluble molecule that binds to RANK. RANK and RANKL are expressed by different cell types and their interaction may modulate immunity by different mechanisms. RANKL/RANK is involved in cross-talk between the bone and the immune system, causing osteoclasts to function as antigen-presenting cells. RANKL is expressed on TILs and Tregs. These cells may stimulate cancer cell metastasis. In contrast, RANK is expressed on tumor-associated macrophages (TAMs) and NK cells [184]. TAMs are generally of the M2 type and favor an immunosuppressive microenvironment, including angiogenesis and tumor growth. RANK–RANKL interaction may promote Treg proliferation, attract macrophages, promote tumor growth and metastasis, angiogenesis and tumor cell stemness [185].

A favorable association between anti-RANKL antibody denosumab and anti-CTLA-4 ipililumab was noted by chance in a case study of a melanoma patient co-administered these two drugs [186]. The likely synergistic effect of this specific combination was subsequently suggested in a retrospective study of patients with metastatic melanoma [187]. The efficacy of this combination was further demonstrated in mouse models of solid tumors [188]. Lymphocytes (in particular, CD8 T cells and NK cells) as well as FcγRs were required for efficacy in this model, with the Fc-competent anti-CTLA-4 being more active that the Fc-silent version. In contrast, the Fc of the anti-RANKL antibody was not required. These encouraging results have led to the opening of three Phase Ib/II clinical trials combining anti-RANKL denosumab with anti-PD-1 nivolumab alone or with anti-CTLA-4 ipililumab (in NSCLC and renal cancers) (Table 6), whose results are awaited. RANK/RANKL inhibition has multiple effects on the immune environment and how these effects may synergize with anti-CTLA-4, anti-PD-1 or other ICI antibodies still needs to be clarified. As for other combinations, synergy or other mechanisms may be context-dependent. 

### 7.8. Combination of ICI and Anti-Angiogenic Antibodies

Antibodies targeting angiogenic factors have long been employed in immunotherapy of solid cancers, with the knowledge that angiogenesis is a factor promoting cancer cell growth. Nonetheless, anti-angiogenic antibodies, combined with chemotherapy, have had limited success so far [189]. This has led to the hypothesis that they could be combined with ICI antibodies, thus targeting different pathways that promote tumor growth. Several clinical trials combining antibodies against angiogenic factors or their receptors and ICI have therefore been started, especially in the context of solid tumors (Table 6). 

### 7.9. Combination with Antibodies Targeting the Complement System

The complement system is an essential component of innate immunity. It is composed of more than 50 soluble, membrane-bound and intracellular proteins, organized as enzyme cascades and their regulators. Complement is activated via 3 different pathways, the classical pathway induced by C1q binding to antibodies or dying cell components, the lectin pathway activated by lectin binding proteins, and the alternative pathways, which amplifies the first two [190]. In the last 10 years, complement has emerged as an important regulator of immunity against tumors, in addition to its well-known role as a sensor and mechanism of elimination of pathogens [191,192,193]. Complement components both activate and recall immune cells (T cells, neutrophils, monocytes, macrophages, etc.) within tumors, most prominently through the C3a and C5a fragments which are potent anaphylatoxins. However, several complement components also dampen immunity, presumably as a negative feedback mechanism to avoid tissue damage by excessive immune response. Several components are implicated in these negative effects of complement, mediating the induction of tolerogenic signals, the recall of C5aR-positive myeloid-derived suppressor cells (MDSC), the induction of PD-L1 on monocytes, the recall and differentiation of immunosuppressive M2-type macrophages within tumors, the activation of Tregs, as well as others [192]. Since C3 and C5 and their receptors are central to these effects, the therapeutic efficacy of combining anti-PD-1 with C5aR or C3aR blockers has been tested in pre-clinical models with interesting results [194,195], leading to the initiation of a clinical trial of the combined treatment of solid tumors with anti-C5aR antibody and anti-PD-1 durvalumab (NCT03665129). Results are awaited.

### 7.10. Combining Different Specificities Using Bispecific or Two Monospecific Antibodies?

Most of the antibody combinations described in the previous paragraphs could be designed as MAb combinations or as BsAbs. Given the different possible formats of bispecifics [26], the latter may be monovalent for each target antigen or bivalent, allowing for greater avidity in the latter case, equivalent to that of the parent MAbs (Figure 2). Whether these differences may have an impact on efficacy, dosing or toxicity is still a fully open question. 

There are advantages and disadvantages of the MAb combinations versus BsAb approaches. Whereas combining two separate MAbs targeting different ICI/immunostimulatory molecules allows fine tuning of the concentration of each, blocking two ICIs in a bispecific format can have the advantage that a single agent is administered, potentially reducing costs for production and treatment. However, using a BsAb against 2 ICI/immunostimulatory molecules does not allow for flexibility in relative dosing. In this case, one cannot taper one MAb in case of toxicity nor modulate/optimize the treatment regimen, for example by treating patients with each target specificity in succession, or at modified dosages. Most current clinical trials are testing the combination of two or more ICI MAbs, of which at least one is already approved, rather than BsAbs. This is explained by the fact that the best combinations still need to be established and this approach allows testing of different combinations relatively rapidly. It is likely that future development will include optimized bispecific formats to target more than one ICI/immune stimulators. Indeed, several BsAbs targeting 2 ICI have been developed and some have reached phase I clinical trials [196,197,198,199].

Other types of combinations, most notably those combining antibodies against a tumor antigen and an ICI, may be more favorable in a bispecific format, since the tumor antigen should be specifically expressed or overexpressed in the tumor, allowing for greater specificity. Therefore, a tumor antigen × ICI BsAb should allow a better localization of the ICI blocking function to the tumor, thanks to the anti-tumor moiety. In addition, in the case of fully competent IgG1 Fc-bearing BsAbs targeting a tumor antigen and an ICI, the drug should have the capacity to activate immunity through both its Fc and its ICI blocking moiety, potentially resulting in enhanced and localized anti-tumor immune responses, involving both innate and adaptive immunity. This kind of approach is in pre-clinical development by different groups [200,201,202].

Another aspect to consider when designing MAb combination versus BsAb strategies is the pharmacokinetics and immunogenicity of the drugs. MAbs are “natural” molecules with little immunogenicity and bear an Fc, which allows a prolonged stability in vivo (generally a half-life of 2–3 weeks). BsAb structures are very variable (Figure 2) and some may contain unnatural peptides, such as linkers or dimerization modules that join different elements or mutations, orscFvs that are less stable than Fabs. These artificial elements may be more immunogenic and may lead to the formation of anti-drug antibodies in vivo [203]. In addition, BsAbs that lack an Fc have a limited half-life in vivo. Others have natural IgG-like structures, including an Fc, thus providing a better framework for stability in vivo [26,204,205].

These aspects emphasize the need for careful design of novel strategies that take into account all aspects which may impact on the efficacy and safety of the drugs.

## 8. Conclusions and Future Prospects

**Figure 3:**
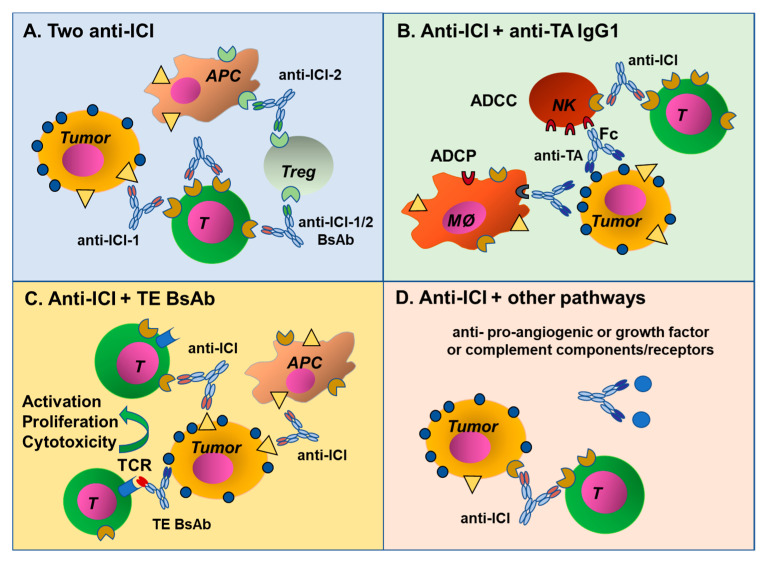
Mechanisms of antibody combinations. (**A**) Antibody combinations targeting two ICI, either as single MAbs or BsAbs. (**B**) Two MAbs targeting an ICI and a tumor antigen, these can also be combined in a BsAb format. (**C**) Combination of a MAb targeting an ICI with a TE BsAb and (**D**) an ICI antibody can be combined with MAbs blocking different pathways involved in tumor growth and metastasis, such as antibodies blocking angiogenic or growth factors, or complement factors and their receptors.

Antibodies targeting ICI have shown impressive efficacy in several solid cancers, especially melanoma. However, effectiveness is very much patient-dependent and for most tumors, ICI antibodies are not sufficient as monotherapy and need to be combined with chemotherapy, radiotherapy, targeted drugs or other therapeutic antibodies. Different combinations of antibodies have been proposed in a variety of types of cancer, with the hope of either inducing a stronger and more specific anti-tumor immune response, or of blocking different and synergistic pathways of tumor growth. Anti-CTLA-4 has been combined with anti-PD-1 and PD-L1, showing that in some contexts, this combination is more effective than monotherapy or chemotherapy. Novel combinations are also being investigated. Figure 3 summarizes the different types and mechanisms of antibody combinations that are being investigated. Clinical data from most studies are still awaited, so conclusions about efficacy of the different combinations are not yet possible. The next challenge will be to define the best treatment strategy for each type of tumor and to identify biomarkers predicting efficacy and/or toxicity.

We believe that ICI expression studies, the sensitivity of different tumors to ICI antibody monotherapy, in vivo animal pre-clinical models, but also a plausible biological rationale, the pharmacodynamic and pharmacokinetic aspects are all important elements to guide the choice of the best combinations of MAbs or novel BsAbs. 

Another important theme for the future will be to define the choice of best doses and regimens for each antibody combination (concurrent versus sequential treatment, etc.), in order to optimize the ratio between response and toxicity. Careful pharmacokinetic and pharmacodynamic analyses will no doubt contribute to optimize treatment schedules. Indeed, specific timing of each intervention is likely to crucially affect the response in the complex interplay of immune cells with each other and with the tumor microenvironment. Finally, the possible occurrence of anti-drug antibodies that may affect the pharmacokinetics and efficacy of single or combined ICI MAbs or BsAbs is an issue that needs further careful investigation in order to optimize these treatments [206].

## Figures and Tables

**Table 1:** Abbreviations.

Abbreviation	Referring to	Abbreviation	Referring to
**A2a**	Adenosine A2a receptor	**HMGB1**	High-mobility group box 1 protein
**A2b**	Adenosine A2b receptor	**HNSCC**	Head and neck squamous cell carcinoma
**ADC**	Antibody-drug conjugate	**ICI**	Immune checkpoint inhibitor
**ADCC**	Antibody dependent cellular cytotoxicity	**ICOS**	Inducible T cell costimulator
**ADCP**	Antibody dependent cellular phagocytosis	**ITIM**	Immunoreceptor typosine based motif
**AML**	Acute myeloid leukemia	**LAG-3**	Lymphocyte activation gene 3
**APC**	Antigen presenting cell	**MAb**	Monoclonal antibody
**ATLL**	Adult T-cell leukemia/lymphoma	**MCC**	Merkel Cell carcinoma
**B-NHL**	B-Non Hodgkin’s lymphoma	**MDSC**	Myeloid derived suppressor cell
**BsAb**	Bispecific antibody	**NSCLC**	Non-Small Cell Lung Cancer
**BTLA**	B and T lymphocyte attenuator	**ORR**	Overall response rate
**cHL**	Classical Hodgkin’s lymphoma	**OS**	Overall survival
**CML**	Chronic myelogenous leukemia	**PD-1**	Programmed cell death protein 1
**CRC**	Colorectal cancer	**PD-L1/2**	PD-1 ligand 1 or 2
**CTLA-4**	Cytotoxic T-lymphocyte-associated protein 4	**PFS**	Progression-free survival
**DART**	Dual affinity retargeting (BsAb format)	**PMBCL**	Primary mediastinal B cell lymphoma
**DC**	Dendritic cell	**PTCL**	Peripheral T cell lymphoma
**DLBCL**	Diffuse large B cell lymphoma	**PVRIG**	Poliovirus receptor -related Ig domain
**DNAM-1**	DNAX accessory protein 1	**RANK(L)**	Receptor activator of nuclear factor kappa-Β (ligand)
**DOR**	Duration of response	**RCC**	Renal cell carcinoma
**EMA**	European Medicines Agency	**RFS**	Relapse-free survival
**EGFR**	Epidermal growth factor receptor	**scFv**	Single chain fragment variable
**EpCAM**	Epithelial Cell Adhesion Molecule	**SCLC**	Small cell lung cancer
**Fab**	Fragment antigen binding	**TAM**	Tumor associated macrophage
**FL**	Follicular Lymphoma	**TCR**	T cell receptor
**FDA**	Food and Drug Administration	**TE**	T cell engaging
**Fv**	Fragment variable (domain)	**TIGIT**	T cell immunoreceptor with immunoglobulin and ITIM domains
**FcγR**	Fc gamma receptor	**TIL**	Tumor infiltrating lymphocyte
**FcRn**	Neonatal Fc receptor	**Treg**	regulatory T cell
**GITR**	Glucocorticoid-induced TNFR-related protein	**UC**	Urothelial carcinoma
**HCC**	Hepatocellular carcinoma	**VEGFR2**	Vascular endothelial growth factor receptor 2
**HCL**	Hairy cell leukemia	**VISTA**	V-domain immunoglobulin suppressor of T-cell activation
**HER2**	Human epidermal growth factor receptor 2		

**Table 2:** Approved unconjugated anti-cancer MAbs (as of 1 May 2020).

Name	Target Antigen	Antibody Type ^a^	First Indication	Year of First Approval ^b^
Rituximab	CD20	Chimeric IgG1k	B-NHL	1997 (US) 1998 (EU)
Ofatumumab	CD20	Human IgG1k	CLL	2009 (US) 2010 (EU)
Obinutuzumab	CD20	Humanized IgG1k; Glycoengin	CLL	2013 (US) 2014 (EU)
Trastuzumab	HER2	Humanized IgG1k	Breast cancer	1998 (US) 2000 (EU)
Pertuzumab	HER2	Humanized IgG1k	Breast cancer	2012 (US) 2013 (EU)
Cetuximab	EGFR	Chimeric IgG1k	CRC	2004 (US/EU)
Panitumumab	EGFR	Human IgG2k	CRC	2006 (US) 2007 (EU)
Necitumumab	EGFR	Human IgG1k	NSCLC	2015 (US/EU)
Daratumumab	CD38	Human IgG1k	MM	2015 (US) 2016 (EU)
Isatuximab	CD38	Chimeric IgG1k	MM	2020 (US)
Alemtuzumab	CD52	Humanized IgG1k	CLL	2001 (US/EU)
Mogamulizumab	CCR4	Humanized IgG1k	T leukemia/lymphoma	2012 Japan 2018 EU
Elotuzumab	SLAMF7	Humanized IgG1k	MM	2015 (US) 2016 (EU)
Olaratumab	PDGRFα	Human IgG1k	Soft tissue sarcoma	2016 (US/EU)
Dinutuximab-β	GD2	Chimeric IgG1k	Neuroblastoma	2015 (US/EU)
Ramucirumab	VEGFR2	Human IgG1k	Gastric cancer	2014 (US/EU)
Bevacizumab	VEGF	Humanized IgG1k	CRC	2004 (US) 2005 (EU)
Tremelimumab	CTLA-4	Human IgG2k	Melanoma	Orphan 2006
Ipilimumab	CTLA-4	Human IgG1k	Melanoma	2011 (US/EU)
Nivolumab	PD-1	Human IgG4k S228P	Melanoma + Solid cancer	2014 (US) 2015 (EU)
Pembrolizumab	PD-1	Humanized IgG4k S228P	Melanoma + Solid cancer + HL + PMBCL	2014 (US) 2015 (EU)
Cemiplimab	PD-1	Human IgG4k S228P	Cutaneous squamous cell carcinoma	2018 (US) 2019 (EU)
Avelumab	PD-L1	Human IgG1k	MC, UC, RCC	2017 (US/EU)
Atezolizumab	PD-L1	Humanized IgG1k mut	UC, NSCLC	2016 (US) 2017 (EU)
Durvalumab	PD-L1	Human IgG1k mut	NSCLC	2017 (US) 2018 (EU)

^a^ IgG4 S228P has mutations to avoid heavy chain exchange; IgG1k mut antibodies have a mutation that render the Fc silent. ^b^ US Federal Drug Administration (FDA) and/or European Medicines Agency (EMA) approval.

**Table 3:** Approved anti-cancer antibody conjugated to drugs, radionuclide and bispecifics.

Name	Target Antigen	Antibody Type	First Indication	Year of Approval
**Antidoby drug conjugates (ADCs)**				
Gemtuzumab ozogamicin	CD33	Humanized IgG4k calicheamicin	AML	2000–2017 (US) 2018 (EU)
Brentuximab vedotin	CD30	Chimeric IgG1k-MMAE	HD and CD30^+^ PTCL	2011 (US) 2012 (EU)
Trastuzumab emtansine	HER2	Humanized IgG1k	Breast	2013 (US/EU)
Inotuzumab ozogamicin	CD22	Humanized IgG4k-calicheamicin	pre-B ALL	2017 (US/EU)
Moxetumomab pasudotox	CD22	Murine IgG1 dsFv Pseudomonas exotoxin	HCL	2018 (US)
Enfortumab vedotin	Nectin-4	Human IgG1k-MMAE	UC	2019 (US)
Polatuzumab vedotin	CD79b	Humanized IgG1k-MMAE	DLBCL	2019 (US) 2020 (EU)
Sacituzumab govitecan	TROP2	Humanized IgG1k-SN-38	Triple-negative breast cancer	2020 (US)
**Radiolabelled Abs**				
Ibritumomab tiuxetan	CD20	Mouse IgG1-Y90	B-NHL	2002 (US) 2004 (EU)
Tositumomab-I131	CD20	Mouse IgG2a-I131	B-NHL	2003 (US)
**Bispecific antibodies (BsAbs)**				
Catumaxomab	EPCAM/CD3	Rat/mouse bispecific mAb	Malignant ascites	2009 (EU)
Blinatumomab	CD19/CD3	Tandem scFv, Bispecifc	ALL	2014 (US) 2015 (EU)

**Table 4:** Major immune checkpoint inhibitory and stimulatory proteins.

Immune Checkpoint Receptor	CD Number	Receptor Family	Cellular Expression of the Receptor	Ligand	CD Number	Cellular Expression of the Ligand
**A. Immune Checkpoint Inhibitory Proteins**						
CTLA-4	CD152	CD28	Activated T-cells and Tregs	CD80	CD80	APC
				CD86	CD86	
PD-1	CD279	CD28	Activated T and B-cells, NK cells and APCs	PD-L1	CD274	Activated DC, Macrophage and Tumors
				PD-L2	CD273	APCs
BTLA	CD272	CD28	T and B-cells, Macrophages, DCs and NK cells	HVEM	CD270	T-cells and Macrophage
LAG3	CD223	-	Activated T-cells, Tregs, B cells, NK cells and Plasmacytoid DCs	MHC class II/Lectins	-	APC
TIGIT	-	CD28	Activated T-cells, Tregs and NK cells	PVR	CD155	DC, APCs and Tumors
				Nectin-2 (PVRL2)	CD112	
TIM3	CD366	-	Activated T-cells (Th1 cells), Treg	Gal9	-	Variety of tissues
				PtdSer		
				HMGB1		
				CEACAM-1		
VISTA (B7-H5)	-	CD28	Macrophages, DCs, Naïve CD4^+^ T-cells, Tregs, Circulating Neutrophils and Monocytes	VSIG-3	-	Neurons and glial cells
NKG2A	CD94	NKG2	NK	HLA-E	-	-
ecto-5′NT	CD73	Ecto-nucleotidase	Many cell types, upregulated in Treg	-	-	-
NTPDase1	CD39	Ecto-nucleotidase	Many cell types, upregulated in Treg	-	-	-
CD47	CD47	Ig superfamily	Ubiquitous	SIRPα	CD172α	Myeloid, neurons
				THBS1 (TSP-1)	-	Extracellular matrix
**B. Immune Checkpoint Stimulatory Proteins**						
CD27	CD27	TNFR	Activated T-cells, B-cells and NK cells	CD70	CD70	Activated T, B-cells and DC
CD28	CD28	CD28	T-cells	B7	CD80	APC
					CD86	
GITR	CD357	TNFR	Tregs and Naïve and Memory T-cells	GITRL	-	DC, Macrophage and Activated B-cells
ICOS	CD278	B7/CD28	Activated T-cells	ICOSLG	CD275	B-cells, Macrophage and DC
NKG2D	CD314	NKG2	NK cells, CD8+ T-cells and γδ T-cells	MHC class I	-	Epithelial and endothelial cells
				UL16-binding protein		
OX40	CD134	TNFR	Activated T cells, Tregs and NK cells	OX40L	CD252	DC, Macrophage, B-cell and Endothelial cells
4-1BB	CD137	TNFR	Activated T and NK cells	4-1BBL	CD137L	DC, Macrophage and B-cells

**Table 5:** Summary of main Phase II–III trials of anti-CTLA-4/PD-1/PD-L1 antibody in combination.

Cancer Type	Trial	Number of Patients	Main Clinical Results		References
**Combination of nivolumab (anti-PD-1) and ipililumab (anti-CTLA-4)**					
Metastatic and Unresectable Melanoma	Phase II NCT01927419	142	**Placebo + Ipilimumab (3 mg/kg)**	2-year OS: 53.6% ORR: 10.6% PFS: 3.0 months (mo)	[131,132]
			**Nivolumab (1 mg/kg) + Ipilimumab (3 mg/kg)**	2-year OS: 63.8% ORR: 55.9% PFS: Not reached	
	Phase III NCT01844505	1296	**Nivolumab (3 mg/kg)**	ORR: 45.0% OS: 36.9 mo PFS: 6.9 mo	[133,134]
			**Ipilimumab (3 mg/kg)**	ORR: 19.0% OS: 19.9 mo PFS: 2.9 mo	
			**Nivolumab (1 mg/kg) + Ipilimumab (3 mg/kg)**	ORR: 58.0% OS: >60 mo PFS: 11.5 mo	
Renal Cell Carcinoma	Phase III NCT02231749	1390	**Nivolumab (3 mg/kg) + Ipilimumab (1 mg/kg)**	ORR: 42.0% OS: Not reached PFS: 8.2 mo	[135,136]
			**Sunitinib (50 mg)**	ORR: 29.0% OS: 26.6 mo PFS: 8.3 mo	
Non-Small-Cell Lung Cancer	Phase III NCT02477826	2220	**Nivolumab (3 mg/kg) + Ipilimumab (1 mg/kg)**	ORR: 45.3% 1-year PFS: 42.6% PFS: 7.2 mo	[141,142]
			**Chemotherapy**	ORR: 26.9% 1-year PFS: 13.2% PFS: 5.5 mo	
Sarcoma	Phase II NCT02500797	96	**Nivolumab (3 mg/kg)**	ORR: 5.0% PFS: 1.7 mo OS: 10.7 mo	[140]
			**Nivolumab (3 mg/kg) + Ipilimumab (1 mg/kg)**	ORR: 16.0% PFS: 4.1 mo OS: 14.3 mo	
Colorectal Cancer	Phase II NCT02060188	183	**Nivolumab (3 mg/kg)**	ORR: 31.1% 1-year OS: 73.4% 1-year PFS: 50.4%	[137,138]
			**Nivolumab (3 mg/kg) + Ipilimumab (1 mg/kg)**	ORR: 54.6% 1-year OS: 85.0% 1-year PFS: 71.0%	
Esophagogastric Cancer	Phase I/II NCT01928394	160	**Nivolumab (3 mg/kg)**	ORR: 12.0% PFS: 1.4 mo OS: 6.2 mo	[139]
			**Nivolumab (1 mg/kg)+ Ipilimumab (3 mg/kg)**	ORR: 24.0% PFS: 1.4 mo OS: 6.9 mo	
			**Nivolumab (3 mg/kg) + Ipilimumab (1 mg/kg)**	ORR: 4.0% PFS: 1.6 mo OS: 4.8 mo	
Recurrent Small-Cell Lung Cancer	Phase I/II (NCT01928394)	243	**Nivolumab (3 mg/kg)**	ORR: 11.6% OS: 5.7 mo PFS: 1.4 mo	[143]
			**Nivolumab (1 mg/kg)+ Ipilimumab (3 mg/kg)**	ORR: 21.9% OS: 4.7 mo PFS: 1.5 mo	
		216	**Nivolumab (3 mg/kg)**	ORR: 10.0%	[144]
			**Nivolumab (1 mg/kg)+ Ipilimumab (3 mg/kg)**	ORR: 23.0%	
			**Nivolumab (3 mg/kg)+ Ipilimumab (1 mg/kg)**	ORR: 19.0%	
Relapsed Malignant Pleural Mesothelioma	Phase II NCT02716272	125	**Nivolumab (3 mg/kg)**	12-week DC: 40.0% ORR: 19.0% PFS: 4.0 mo OS: 11.9 mo	[145]
			**Nivolumab (3 mg/kg)+ Ipilimumab (1 mg/kg)**	12-week DC: 52.0% ORR: 28.0% PFS: 5.6 mo OS: 15.9 mo	
**Combination of durvalumab (anti-PD-1) and tremelimumab (anti-CTLA-4)**					
Squamous Cell Carcinoma of the Head and Neck	Phase II randomized NCT02319044	267	**Durvalumab (10 mg/kg)**	ORR: 9.2% PFS: 1.9 mo OS: 6.0 mo	[146,147]
			**Tremelimumab (10 mg/kg)**	ORR: 1.6% PFS: 1.9 mo OS: 5.5 mo	
			**Durvalumab (20 mg/kg) + Tremelimumab (1 mg/kg)**	ORR: 7.8% PFS: 2.0 mo OS: 7.6 mo	
	Phase III NCT02369874	736	**Durvalumab (10 mg/kg)**	ORR: 17.9% PFS: 2.1 mo OS: 7.6 mo	[148]
			**Durvalumab (20 mg/kg) + Tremelimumab (1 mg/kg)**	ORR: 18.2% PFS: 2.0 mo OS: 6.5 mo	
			**Chemotherapy**	ORR: 17.3% PFS: 3.7 mo OS: 8.3 mo	
NSCLC	Phase III NCT02453282	1118	**Durvalumab (20 mg/kg)**	OS: 12.3 mo PFS: 2.8 mo	[150]
			**Durvalumab (20 mg/kg) + Tremelimumab (1 mg/kg)**	OS: 11.2 mo PFS: 9.9 mo	
			**Chemotherapy**	OS: 11.8 mo PFS: 5.4 mo	
Metastatic Pancreatic Ductal Adenocarcinoma	Phase IINCT02558894	65	**Durvalumab (1.5 g)**	ORR: 0.0% PFS: 1.5 mo OS: 3.6 mo	[149]
			**Durvalumab (1.5 g) + Tremelimumab (75 mg)**	ORR: 3.1% PFS: 1.5 mo OS: 3.1 mo	
**Combination of pembrolizumab (anti-PD-1) and trastuzumab (anti-HER2)**					
Advanced Metastatic Breast Cancer (trastuzumab resistant)	Phase I/II NCT02129556	52 (Onlyphase II: 40 PDL1+, 12 PDL1−)	Pembrolizumab (200 mg) + Trastuzumab (6 mg/kg)	**ORR:** PD-L1+: 15.0% PD-L1−: 0.0%	[98]
				**OS at 12 months:** PD-L1+: 65.0% PD-L1−: 12.0%	
				**PFS:** PD-L1+: 2.7 mo PD-L1−: 2.5 mo	

**Table 6:** Combinations of MAbs/BsAbs being tested in Phase I and II clinical trials.

First Specificity	Combined with Antibodies Against	Category of Combination	Diseases	References
PD-1 or PD-L1	TIGIT	2 ICI	Solid tumors and hematological malignancies	[43] [154] [155] [160] [166] [177] [183] [193]
	TIM3			
	LAG3			
	NKG2A			
	CD73			
	PVRIG			
	CD47			
	CD137	ICI + immune stimulator		
	OX40			
	CD27			
	GITR			
	EGFR	ICI + anti-tumor MAb		
	HER2			
	CD20			
	CD22			
	CCR4			
	FGFR3			
	CD19 × CD3 ± CTLA4	ICI + TE BsAb		
	CD20 × CD3			
	gPA × CD3			
	VEGF × ANG2	ICI + anti-angiogenic BsAb		
	C5aR	ICI + anti-complement receptor		
	TGF-β RII	Miscellaneous		
	RANKL ± CTLA4			
CTLA4	LAG3	2 ICI	Melanoma	[152]
	OX40	ICI + immune stimulator	Solid tumors	[169]
	EGFR	ICI + anti-tumor MAb	HNSCC	[177]
OX40	CTLA-4 ± PD-1	2 ICI + immune stimulator	Solid tumors	[166]
	CD137	2 immune stimulator	Solid tumors	
	TLR4 ot TLR9			
	CD20	immune stimulator + anti-tumor MAb	DLBCL	
CD27	GPNMB (ADC)	immune stimulator and ADC	Melanoma	[166]
CD137	CCR4	immune stimulator and anti-tumor MAb	Advanced solid tumors	[166,199]
	CD20 ± PD-L1		DLBCL	
	EGFR		CRC	
	HER2 (Mab or ADC)		Breast cancer	
	OX40+PD-L1	2 immune stimulators and 1 ICI antibody	Solid tumors	
CD47	EGFR	ICI + anti-tumor MAb	Colorectal	[162]
	CD20		B-NHL	
NKG2A	EGFR	ICI + anti-tumor MAb	Squamous cell carcinoma of head and neck	[157]
CD73	EGFR	ICI + anti-tumor MAb	Solid tumors	[160]
LAG3	PD-1 + TIM3	3 ICI antibodies	Solid + Lymphoma	[152]
	PD-1 + CTLA4 ± CD38	3 ICI MAbs + anti-tumor MAb	Advance tunors	
	PD-1 + CD137	2 ICI MAbs + 1 immune stimulator	Glioblastoma	
GITR	PD-1 ± CTLA4	2 ICI MAbs + 1 immune stimulator	Solid tumors	[166]

## Supplementary Materials

The following are available online at https://www.mdpi.com/2073-4468/9/2/17/s1, Table S1: Results of major Phase II and III clinical trials using anti-ICI as monotherapy.

## References

[1] Golay J. (2017). Direct targeting of cancer cells with antibodies: What can we learn from the successes and failure of unconjugated antibodies for lymphoid neoplasias?. J. Autoimmun..

[2] Weiner G.J. (2015). Building better monoclonal antibody-based therapeutics. Nat. Rev. Cancer.

[3] Taylor R.P., Lindorfer M.A. (2016). Cytotoxic mechanisms of immunotherapy: Harnessing complement in the action of anti-tumor monoclonal antibodies. Semin. Immunol..

[4] Sedykh S.E., Prinz V.V., Buneva V.N., Nevinsky G.A. (2018). Bispecific antibodies: Design, therapy, perspectives. Drug Des. Devel. Ther..

[5] Gajewski T.F., Schreiber H., Fu Y.-X. (2013). Innate and adaptive immune cells in the tumor microenvironment. Nat. Immunol..

[6] Ansell S.M., Vonderheide R.H. (2013). Cellular composition of the tumor microenvironment. Am. Soc. Clin. Oncol. Educ. Book.

[7] Guerrouahen B.S., Maccalli C., Cugno C., Rutella S., Akporiaye E.T. (2020). Reverting Immune Suppression to Enhance Cancer Immunotherapy. Front. Oncol..

[8] Sanmamed M.F., Pastor F., Rodriguez A., Perez-Gracia J.L., Rodriguez-Ruiz M.E., Jure-Kunkel M., Melero I. (2015). Agonists of Co-stimulation in Cancer Immunotherapy Directed Against CD137, OX40, GITR, CD27, CD28, and ICOS. Semin. Oncol..

[9] Waldmann T.A. (2018). Cytokines in Cancer Immunotherapy. Cold Spring Harb. Perspect. Biol..

[10] Neri D. (2019). Antibody-Cytokine Fusions: Versatile Products for the Modulation of Anticancer Immunity. Cancer Immunol. Res..

[11] Webb E.S., Liu P., Baleeiro R., Lemoine N.R., Yuan M., Wang Y. (2018). Immune checkpoint inhibitors in cancer therapy. J. Biomed. Res..

[12] Li X., Shao C., Shi Y., Han W. (2018). Lessons learned from the blockade of immune checkpoints in cancer immunotherapy. J. Hematol. Oncol..

[13] Kennedy L.B., Salama A.K.S. (2020). A review of cancer immunotherapy toxicity. CA Cancer J. Clin..

[14] Das S., Johnson D.B. (2019). Immune-related adverse events and anti-tumor efficacy of immune checkpoint inhibitors. J. Immunother. Cancer.

[15] McGonagle D., Bragazzi N.L., Amital H., Watad A. (2020). Mechanistic classification of immune checkpoint inhibitor toxicity as a pointer to minimal treatment strategies to further improve survival. Autoimmun. Rev..

[16] Saunders K.O. (2019). Conceptual Approaches to Modulating Antibody Effector Functions and Circulation Half-Life. Front. Immunol..

[17] Kang T.H., Jung S.T. (2019). Boosting therapeutic potency of antibodies by taming Fc domain functions. Exp. Mol. Med..

[18] Salfeld J.G. (2007). Isotype selection in antibody engineering. Nat. Biotechnol..

[19] De Aguiar R.B., de Moraes J.Z. (2019). Exploring the Immunological Mechanisms Underlying the Anti-vascular Endothelial Growth Factor Activity in Tumors. Front. Immunol..

[20] Bournazos S., Wang T.T., Dahan R., Maamary J., Ravetch J.V. (2017). Signaling by Antibodies: Recent Progress. Annu. Rev. Immunol..

[21] Davies A.M., Sutton B.J. (2015). Human IgG4: A structural perspective. Immunol. Rev..

[22] Alsaab H.O., Sau S., Alzhrani R., Tatiparti K., Bhise K., Kashaw S.K., Iyer A.K. (2017). PD-1 and PD-L1 Checkpoint Signaling Inhibition for Cancer Immunotherapy: Mechanism, Combinations, and Clinical Outcome. Front. Pharmacol..

[23] Chen X., Song X., Li K., Zhang T. (2019). FcγR-Binding Is an Important Functional Attribute for Immune Checkpoint Antibodies in Cancer Immunotherapy. Front. Immunol..

[24] Challa D.K., Velmurugan R., Ober R.J., Sally Ward E. (2014). FcRn: From molecular interactions to regulation of IgG pharmacokinetics and functions. Curr. Top. Microbiol. Immunol..

[25] Stapleton N.M., Einarsdóttir H.K., Stemerding A.M., Vidarsson G. (2015). The multiple facets of FcRn in immunity. Immunol. Rev..

[26] Spiess C., Zhai Q., Carter P.J. (2015). Alternative molecular formats and therapeutic applications for bispecific antibodies. Mol. Immunol..

[27] Carter P.J., Lazar G.A. (2018). Next generation antibody drugs: Pursuit of the “high-hanging fruit”. Nat. Rev. Drug. Discov..

[28] Brinkmann U., Kontermann R.E. (2017). The making of bispecific antibodies. MAbs.

[29] Goebeler M.-E., Bargou R.C. (2020). T cell-engaging therapies—BiTEs and beyond. Nat. Rev. Clin. Oncol..

[30] Bargou R., Leo E., Zugmaier G., Klinger M., Goebeler M., Knop S., Noppeney R., Viardot A., Hess G., Schuler M. (2008). Tumor regression in cancer patients by very low doses of a T cell-engaging antibody. Science.

[31] Dobosz P., Dzieciątkowski T. (2019). The Intriguing History of Cancer Immunotherapy. Front. Immunol..

[32] Fritz J.M., Lenardo M.J. (2019). Development of immune checkpoint therapy for cancer. J. Exp. Med..

[33] Ribatti D. (2016). The concept of immune surveillance against tumors: The first theories. Oncotarget.

[34] Dunn G.P., Bruce A.T., Ikeda H., Old L.J., Schreiber R.D. (2002). Cancer immunoediting: From immunosurveillance to tumor escape. Nat. Immunol..

[35] Schreiber R.D., Old L.J., Smyth M.J. (2011). Cancer immunoediting: Integrating immunity’s roles in cancer suppression and promotion. Science.

[36] Feinberg A.P., Ohlsson R., Henikoff S. (2006). The epigenetic progenitor origin of human cancer. Nat. Rev. Genet..

[37] Spranger S., Gajewski T.F. (2018). Impact of oncogenic pathways on evasion of antitumour immune responses. Nat. Rev. Cancer.

[38] Reeves E., James E. (2017). Antigen processing and immune regulation in the response to tumours. Immunology.

[39] Binnewies M., Roberts E.W., Kersten K., Chan V., Fearon D.F., Merad M., Coussens L.M., Gabrilovich D.I., Ostrand-Rosenberg S., Hedrick C.C. (2018). Understanding the tumor immune microenvironment (TIME) for effective therapy. Nat. Med..

[40] Driessens G., Kline J., Gajewski T.F. (2009). Costimulatory and coinhibitory receptors in anti-tumor immunity. Immunol. Rev..

[41] Bonavida B., Chouaib S. (2017). Resistance to anticancer immunity in cancer patients: Potential strategies to reverse resistance. Ann. Oncol..

[42] Darvin P., Toor S.M., Sasidharan Nair V., Elkord E. (2018). Immune checkpoint inhibitors: Recent progress and potential biomarkers. Exp. Mol. Med..

[43] Pardoll D.M. (2012). The blockade of immune checkpoints in cancer immunotherapy. Nat. Rev. Cancer.

[44] Freeman G.J., Long A.J., Iwai Y., Bourque K., Chernova T., Nishimura H., Fitz L.J., Malenkovich N., Okazaki T., Byrne M.C. (2000). Engagement of the Pd-1 Immunoinhibitory Receptor by a Novel B7 Family Member Leads to Negative Regulation of Lymphocyte Activation. J. Exp. Med..

[45] Korman A.J., Peggs K.S., Allison J.P. (2006). Checkpoint Blockade in Cancer Immunotherapy. Adv. Immunol..

[46] Walunas T.L., Lenschow D.J., Bakker C.Y., Linsley P.S., Freeman G.J., Green J.M., Thompson C.B., Bluestone J.A. (1994). CTLA-4 can function as a negative regulator of T cell activation. Immunity.

[47] Hodi F.S., O’Day S.J., McDermott D.F., Weber R.W., Sosman J.A., Haanen J.B., Gonzalez R., Robert C., Schadendorf D., Hassel J.C. (2010). Improved survival with ipilimumab in patients with metastatic melanoma. N. Engl. J. Med..

[48] Weber J.S., D’Angelo S.P., Minor D., Hodi F.S., Gutzmer R., Neyns B., Hoeller C., Khushalani N.I., Miller W.H., Lao C.D. (2015). Nivolumab versus chemotherapy in patients with advanced melanoma who progressed after anti-CTLA-4 treatment (CheckMate 037): A randomised, controlled, open-label, phase 3 trial. Lancet Oncol..

[49] Wolchok J.D., Saenger Y. (2008). The mechanism of anti-CTLA-4 activity and the negative regulation of T-cell activation. Oncologist.

[50] Wing J.B., Tanaka A., Sakaguchi S. (2019). Human FOXP3+ Regulatory T Cell Heterogeneity and Function in Autoimmunity and Cancer. Immunity.

[51] Simpson T.R., Li F., Montalvo-Ortiz W., Sepulveda M.A., Bergerhoff K., Arce F., Roddie C., Henry J.Y., Yagita H., Wolchok J.D. (2013). Fc-dependent depletion of tumor-infiltrating regulatory T cells co-defines the efficacy of anti-CTLA-4 therapy against melanoma. J. Exp. Med..

[52] Seidel J.A., Otsuka A., Kabashima K. (2018). Anti-PD-1 and Anti-CTLA-4 Therapies in Cancer: Mechanisms of Action, Efficacy, and Limitations. Front. Oncol..

[53] Boussiotis V.A. (2016). Molecular and Biochemical Aspects of the PD-1 Checkpoint Pathway. N. Engl. J. Med..

[54] Long L., Zhang X., Chen F., Pan Q., Phiphatwatchara P., Zeng Y., Chen H. (2018). The promising immune checkpoint LAG-3: From tumor microenvironment to cancer immunotherapy. Genes Cancer.

[55] Sedy J.R., Gavrieli M., Potter K.G., Hurchla M.A., Lindsley R.C., Hildner K., Scheu S., Pfeffer K., Ware C.F., Murphy T.L. (2005). B and T lymphocyte attenuator regulates T cell activation through interaction with herpesvirus entry mediator. Nat. Immunol..

[56] Das M., Zhu C., Kuchroo V.K. (2017). Tim-3 and its role in regulating anti-tumor immunity. Immunol. Rev..

[57] Friedlaender A., Addeo A., Banna G. (2019). New emerging targets in cancer immunotherapy: The role of TIM3. ESMO Open.

[58] Lines J.L., Sempere L.F., Wang L., Pantazi E., Mak J., O’Connell S., Ceeraz S., Suriawinata A.A., Yan S., Ernstoff M.S. (2014). VISTA is an immune checkpoint molecule for human T cells. Cancer Res..

[59] Solomon B.L., Garrido-Laguna I. (2018). TIGIT: A novel immunotherapy target moving from bench to bedside. Cancer Immunol. Immunother..

[60] Manieri N.A., Chiang E.Y., Grogan J.L. (2017). TIGIT: A Key Inhibitor of the Cancer Immunity Cycle. Trends Immunol..

[61] Chao M.P., Weissman I.L., Majeti R. (2012). The CD47-SIRPα pathway in cancer immune evasion and potential therapeutic implications. Curr. Opin. Immunol..

[62] Brown K.E. (2018). Revisiting CD28 Superagonist TGN1412 as Potential Therapeutic for Pediatric B Cell Leukemia: A Review. Diseases.

[63] Amatore F., Gorvel L., Olive D. (2018). Inducible Co-Stimulator (ICOS) as a potential therapeutic target for anti-cancer therapy. Expert Opin. Ther. Targets.

[64] Knee D.A., Hewes B., Brogdon J.L. (2016). Rationale for anti-GITR cancer immunotherapy. Eur. J. Cancer.

[65] López-Soto A., Huergo-Zapico L., Acebes-Huerta A., Villa-Alvarez M., Gonzalez S. (2015). NKG2D signaling in cancer immunosurveillance. Int. J. Cancer.

[66] Aspeslagh S., Postel-Vinay S., Rusakiewicz S., Soria J.-C., Zitvogel L., Marabelle A. (2016). Rationale for anti-OX40 cancer immunotherapy. Eur. J. Cancer.

[67] Chester C., Sanmamed M.F., Wang J., Melero I. (2018). Immunotherapy targeting 4-1BB: Mechanistic rationale, clinical results, and future strategies. Blood.

[68] Ribas A., Kefford R., Marshall M.A., Punt C.J.A., Haanen J.B., Marmol M., Garbe C., Gogas H., Schachter J., Linette G. (2013). Phase III randomized clinical trial comparing tremelimumab with standard-of-care chemotherapy in patients with advanced melanoma. J. Clin. Oncol..

[69] Maio M., Scherpereel A., Calabrò L., Aerts J., Cedres Perez S., Bearz A., Nackaerts K., Fennell D.A., Kowalski D., Tsao A.S. (2017). Tremelimumab as second-line or third-line treatment in relapsed malignant mesothelioma (DETERMINE): A multicentre, international, randomised, double-blind, placebo-controlled phase 2b trial. Lancet Oncol..

[70] Maio M., Grob J.-J., Aamdal S., Bondarenko I., Robert C., Thomas L., Garbe C., Chiarion-Sileni V., Testori A., Chen T.-T. (2015). Five-year survival rates for treatment-naive patients with advanced melanoma who received ipilimumab plus dacarbazine in a phase III trial. J. Clin. Oncol..

[71] Robert C., Thomas L., Bondarenko I., O’Day S., Weber J., Garbe C., Lebbe C., Baurain J.-F., Testori A., Grob J.-J. (2011). Ipilimumab plus Dacarbazine for Previously Untreated Metastatic Melanoma. N. Engl. J. Med..

[72] Geoerger B., Bergeron C., Gore L., Sender L., Dunkel I.J., Herzog C., Brochez L., Cruz O., Nysom K., Berghorn E. (2017). Phase II study of ipilimumab in adolescents with unresectable stage III or IV malignant melanoma. Eur. J. Cancer.

[73] Eggermont A.M.M., Chiarion-Sileni V., Grob J.-J., Dummer R., Wolchok J.D., Schmidt H., Hamid O., Robert C., Ascierto P.A., Richards J.M. (2016). Prolonged Survival in Stage III Melanoma with Ipilimumab Adjuvant Therapy. N. Engl. J. Med..

[74] Ascierto P.A., Del Vecchio M., Robert C., Mackiewicz A., Chiarion-Sileni V., Arance A., Lebbé C., Bastholt L., Hamid O., Rutkowski P. (2017). Ipilimumab 10 mg/kg versus ipilimumab 3 mg/kg in patients with unresectable or metastatic melanoma: A randomised, double-blind, multicentre, phase 3 trial. Lancet Oncol..

[75] Robert C., Ribas A., Schachter J., Arance A., Grob J.-J., Mortier L., Daud A., Carlino M.S., McNeil C.M., Lotem M. (2019). Pembrolizumab versus ipilimumab in advanced melanoma (KEYNOTE-006): Post-hoc 5-year results from an open-label, multicentre, randomised, controlled, phase 3 study. Lancet Oncol..

[76] Bang Y.-J., Cho J.Y., Kim Y.H., Kim J.W., Di Bartolomeo M., Ajani J.A., Yamaguchi K., Balogh A., Sanchez T., Moehler M. (2017). Efficacy of Sequential Ipilimumab Monotherapy versus Best Supportive Care for Unresectable Locally Advanced/Metastatic Gastric or Gastroesophageal Junction Cancer. Clin. Cancer Res..

[77] Grywalska E., Sobstyl M., Putowski L., Roliński J. (2019). Current Possibilities of Gynecologic Cancer Treatment with the Use of Immune Checkpoint Inhibitors. Int. J. Mol. Sci..

[78] Larkin J., Minor D., D’Angelo S., Neyns B., Smylie M., Miller W.H., Gutzmer R., Linette G., Chmielowski B., Lao C.D. (2018). Overall Survival in Patients With Advanced Melanoma Who Received Nivolumab Versus Investigator’s Choice Chemotherapy in CheckMate 037: A Randomized, Controlled, Open-Label Phase III Trial. J. Clin. Oncol..

[79] Ascierto P.A., Long G.V., Robert C., Brady B., Dutriaux C., Di Giacomo A.M., Mortier L., Hassel J.C., Rutkowski P., McNeil C. (2019). Survival Outcomes in Patients With Previously Untreated BRAF Wild-Type Advanced Melanoma Treated With Nivolumab Therapy. JAMA Oncol..

[80] Herrera A.F., Goy A., Mehta A., Ramchandren R., Pagel J.M., Svoboda J., Guan S., Hill J.S., Kwei K., Liu E.A. (2020). Safety and activity of ibrutinib in combination with durvalumab in patients with relapsed or refractory follicular lymphoma or diffuse large B-cell lymphoma. Am. J. Hematol..

[81] Ansell S.M., Minnema M.C., Johnson P., Timmerman J.M., Armand P., Shipp M.A., Rodig S.J., Ligon A.H., Roemer M.G.M., Reddy N. (2019). Nivolumab for Relapsed/Refractory Diffuse Large B-Cell Lymphoma in Patients Ineligible for or Having Failed Autologous Transplantation: A Single-Arm, Phase II Study. J. Clin. Oncol..

[82] Younes A., Santoro A., Shipp M., Zinzani P.L., Timmerman J.M., Ansell S., Armand P., Fanale M., Ratanatharathorn V., Kuruvilla J. (2016). Nivolumab for classical Hodgkin’s lymphoma after failure of both autologous stem-cell transplantation and brentuximab vedotin: A multicentre, multicohort, single-arm phase 2 trial. Lancet Oncol..

[83] Armand P., Engert A., Younes A., Fanale M., Santoro A., Zinzani P.L., Timmerman J.M., Collins G.P., Ramchandren R., Cohen J.B. (2018). Nivolumab for Relapsed/Refractory Classic Hodgkin Lymphoma After Failure of Autologous Hematopoietic Cell Transplantation: Extended Follow-Up of the Multicohort Single-Arm Phase II CheckMate 205 Trial. J. Clin. Oncol..

[84] Sharma P., Retz M., Siefker-Radtke A., Baron A., Necchi A., Bedke J., Plimack E.R., Vaena D., Grimm M.-O., Bracarda S. (2017). Nivolumab in metastatic urothelial carcinoma after platinum therapy (CheckMate 275): A multicentre, single-arm, phase 2 trial. Lancet Oncol..

[85] Ferris R.L., Blumenschein G., Fayette J., Guigay J., Colevas A.D., Licitra L., Harrington K., Kasper S., Vokes E.E., Even C. (2016). Nivolumab for Recurrent Squamous-Cell Carcinoma of the Head and Neck. N. Engl. J. Med..

[86] Ma B.B.Y., Lim W.-T., Goh B.-C., Hui E.P., Lo K.-W., Pettinger A., Foster N.R., Riess J.W., Agulnik M., Chang A.Y.C. (2018). Antitumor Activity of Nivolumab in Recurrent and Metastatic Nasopharyngeal Carcinoma: An International, Multicenter Study of the Mayo Clinic Phase 2 Consortium (NCI-9742). J. Clin. Oncol..

[87] Rizvi N.A., Mazières J., Planchard D., Stinchcombe T.E., Dy G.K., Antonia S.J., Horn L., Lena H., Minenza E., Mennecier B. (2015). Activity and safety of nivolumab, an anti-PD-1 immune checkpoint inhibitor, for patients with advanced, refractory squamous non-small-cell lung cancer (CheckMate 063): A phase 2, single-arm trial. Lancet Oncol..

[88] Wu Y.-L., Lu S., Cheng Y., Zhou C., Wang J., Mok T., Zhang L., Tu H.-Y., Wu L., Feng J. (2019). Nivolumab Versus Docetaxel in a Predominantly Chinese Patient Population With Previously Treated Advanced NSCLC: CheckMate 078 Randomized Phase III Clinical Trial. J. Thorac. Oncol..

[89] Vokes E.E., Ready N., Felip E., Horn L., Burgio M.A., Antonia S.J., Arén Frontera O., Gettinger S., Holgado E., Spigel D. (2018). Nivolumab versus docetaxel in previously treated advanced non-small-cell lung cancer (CheckMate 017 and CheckMate 057): 3-year update and outcomes in patients with liver metastases. Ann. Oncol..

[90] Reck M., Brahmer J., Bennett B., Taylor F., Penrod J.R., DeRosa M., Dastani H., Spigel D.R., Gralla R.J. (2018). Evaluation of health-related quality of life and symptoms in patients with advanced non-squamous non-small cell lung cancer treated with nivolumab or docetaxel in CheckMate 057. Eur. J. Cancer.

[91] Carbone D.P., Reck M., Paz-Ares L., Creelan B., Horn L., Steins M., Felip E., van den Heuvel M.M., Ciuleanu T.-E., Badin F. (2017). First-Line Nivolumab in Stage IV or Recurrent Non-Small-Cell Lung Cancer. N. Engl. J. Med..

[92] Escudier B., Sharma P., McDermott D.F., George S., Hammers H.J., Srinivas S., Tykodi S.S., Sosman J.A., Procopio G., Plimack E.R. (2017). CheckMate 025 Randomized Phase 3 Study: Outcomes by Key Baseline Factors and Prior Therapy for Nivolumab Versus Everolimus in Advanced Renal Cell Carcinoma. Eur. Urol..

[93] Motzer R.J., Rini B.I., McDermott D.F., Redman B.G., Kuzel T.M., Harrison M.R., Vaishampayan U.N., Drabkin H.A., George S., Logan T.F. (2015). Nivolumab for Metastatic Renal Cell Carcinoma: Results of a Randomized Phase II Trial. J. Clin. Oncol..

[94] Roemer M.G.M., Redd R.A., Cader F.Z., Pak C.J., Abdelrahman S., Ouyang J., Sasse S., Younes A., Fanale M., Santoro A. (2018). Major Histocompatibility Complex Class II and Programmed Death Ligand 1 Expression Predict Outcome After Programmed Death 1 Blockade in Classic Hodgkin Lymphoma. J. Clin. Oncol..

[95] Ratner L., Waldmann T.A., Janakiram M., Brammer J.E. (2018). Rapid Progression of Adult T-Cell Leukemia-Lymphoma after PD-1 Inhibitor Therapy. N. Engl. J. Med..

[96] Rauch D.A., Conlon K.C., Janakiram M., Brammer J.E., Harding J.C., Ye B.H., Zang X., Ren X., Olson S., Cheng X. (2019). Rapid progression of adult T-cell leukemia/lymphoma as tumor-infiltrating Tregs after PD-1 blockade. Blood.

[97] Eggermont A.M.M., Blank C.U., Mandala M., Long G.V., Atkinson V., Dalle S., Haydon A., Lichinitser M., Khattak A., Carlino M.S. (2018). Adjuvant Pembrolizumab versus Placebo in Resected Stage III Melanoma. N. Engl. J. Med..

[98] Loi S., Giobbie-Hurder A., Gombos A., Bachelot T., Hui R., Curigliano G., Campone M., Biganzoli L., Bonnefoi H., Jerusalem G. (2019). Pembrolizumab plus trastuzumab in trastuzumab-resistant, advanced, HER2-positive breast cancer (PANACEA): A single-arm, multicentre, phase 1b-2 trial. Lancet Oncol..

[99] Shah M.A., Kojima T., Hochhauser D., Enzinger P., Raimbourg J., Hollebecque A., Lordick F., Kim S.-B., Tajika M., Kim H.T. (2019). Efficacy and Safety of Pembrolizumab for Heavily Pretreated Patients With Advanced, Metastatic Adenocarcinoma or Squamous Cell Carcinoma of the Esophagus. JAMA Oncol..

[100] Shitara K., Özgüroğlu M., Bang Y.-J., Di Bartolomeo M., Mandalà M., Ryu M.-H., Fornaro L., Olesiński T., Caglevic C., Chung H.C. (2018). Pembrolizumab versus paclitaxel for previously treated, advanced gastric or gastro-oesophageal junction cancer (KEYNOTE-061): A randomised, open-label, controlled, phase 3 trial. Lancet.

[101] Cohen E.E.W., Soulières D., Le Tourneau C., Dinis J., Licitra L., Ahn M.-J., Soria A., Machiels J.-P., Mach N., Mehra R. (2019). Pembrolizumab versus methotrexate, docetaxel, or cetuximab for recurrent or metastatic head-and-neck squamous cell carcinoma (KEYNOTE-040): A randomised, open-label, phase 3 study. Lancet.

[102] Herbst R.S., Baas P., Kim D.-W., Felip E., Pérez-Gracia J.L., Han J.-Y., Molina J., Kim J.-H., Arvis C.D., Ahn M.-J. (2016). Pembrolizumab versus docetaxel for previously treated, PD-L1-positive, advanced non-small-cell lung cancer (KEYNOTE-010): A randomised controlled trial. Lancet.

[103] Reck M., Rodríguez-Abreu D., Robinson A.G., Hui R., Csőszi T., Fülöp A., Gottfried M., Peled N., Tafreshi A., Cuffe S. (2016). Pembrolizumab versus Chemotherapy for PD-L1-Positive Non-Small-Cell Lung Cancer. N. Engl. J. Med..

[104] Mok T.S.K., Wu Y.-L., Kudaba I., Kowalski D.M., Cho B.C., Turna H.Z., Castro G., Srimuninnimit V., Laktionov K.K., Bondarenko I. (2019). Pembrolizumab versus chemotherapy for previously untreated, PD-L1-expressing, locally advanced or metastatic non-small-cell lung cancer (KEYNOTE-042): A randomised, open-label, controlled, phase 3 trial. Lancet.

[105] Balar A.V., Castellano D., O’Donnell P.H., Grivas P., Vuky J., Powles T., Plimack E.R., Hahn N.M., de Wit R., Pang L. (2017). First-line pembrolizumab in cisplatin-ineligible patients with locally advanced and unresectable or metastatic urothelial cancer (KEYNOTE-052): A multicentre, single-arm, phase 2 study. Lancet Oncol..

[106] Fradet Y., Bellmunt J., Vaughn D.J., Lee J.L., Fong L., Vogelzang N.J., Climent M.A., Petrylak D.P., Choueiri T.K., Necchi A. (2019). Randomized phase III KEYNOTE-045 trial of pembrolizumab versus paclitaxel, docetaxel, or vinflunine in recurrent advanced urothelial cancer: Results of >2 years of follow-up. Ann Oncol..

[107] Chen R., Zinzani P.L., Fanale M.A., Armand P., Johnson N.A., Brice P., Radford J., Ribrag V., Molin D., Vassilakopoulos T.P. (2017). Phase II Study of the Efficacy and Safety of Pembrolizumab for Relapsed/Refractory Classic Hodgkin Lymphoma. J. Clin. Oncol..

[108] Migden M.R., Khushalani N.I., Chang A.L.S., Lewis K.D., Schmults C.D., Hernandez-Aya L., Meier F., Schadendorf D., Guminski A., Hauschild A. (2020). Cemiplimab in locally advanced cutaneous squamous cell carcinoma: Results from an open-label, phase 2, single-arm trial. Lancet Oncol..

[109] Wu Y., Chen W., Xu Z.P., Gu W. (2019). PD-L1 Distribution and Perspective for Cancer Immunotherapy—Blockade, Knockdown, or Inhibition. Front. Immunol..

[110] Greillier L., Tomasini P., Barlesi F. (2015). Necitumumab for non-small cell lung cancer. Expert Opin. Biol. Ther..

[111] Weiner L.M., Surana R., Wang S. (2010). Antibodies and cancer therapy: Versatile platforms for cancer immunotherapy. Nat. Rev. Immunol..

[112] Scheuer W., Friess T., Burtscher H., Bossenmaier B., Endl J., Hasmann M. (2009). Strongly enhanced antitumor activity of trastuzumab and pertuzumab combination treatment on HER2-positive human xenograft tumor models. Cancer Res..

[113] Boyerinas B., Jochems C., Fantini M., Heery C.R., Gulley J.L., Tsang K.Y., Schlom J. (2015). Antibody-dependent cellular cytotoxicity (ADCC) activity of a novel anti-PD-L1 antibody avelumab (MSB0010718C) on human tumor cells. Cancer Immunol. Res..

[114] Jardim D.L., de Melo Gagliato D., Giles F.J., Kurzrock R. (2018). Analysis of Drug Development Paradigms for Immune Checkpoint inhibitors. Clin. Cancer Res..

[115] Bang Y.-J., Ruiz E.Y., Van Cutsem E., Lee K.-W., Wyrwicz L., Schenker M., Alsina M., Ryu M.-H., Chung H.-C., Evesque L. (2018). Phase III, randomised trial of avelumab versus physician’s choice of chemotherapy as third-line treatment of patients with advanced gastric or gastro-oesophageal junction cancer: Primary analysis of JAVELIN Gastric 300. Ann. Oncol..

[116] Kaufman H.L., Russell J., Hamid O., Bhatia S., Terheyden P., D’Angelo S.P., Shih K.C., Lebbé C., Linette G.P., Milella M. (2016). Avelumab in patients with chemotherapy-refractory metastatic Merkel cell carcinoma: A multicentre, single-group, open-label, phase 2 trial. Lancet Oncol..

[117] Kaufman H.L., Russell J.S., Hamid O., Bhatia S., Terheyden P., D’Angelo S.P., Shih K.C., Lebbé C., Milella M., Brownell I. (2018). Updated efficacy of avelumab in patients with previously treated metastatic Merkel cell carcinoma after ≥1 year of follow-up: JAVELIN Merkel 200, a phase 2 clinical trial. J. Immunother. Cancer.

[118] Barlesi F., Vansteenkiste J., Spigel D., Ishii H., Garassino M., de Marinis F., Özgüroğlu M., Szczesna A., Polychronis A., Uslu R. (2018). Avelumab versus docetaxel in patients with platinum-treated advanced non-small-cell lung cancer (JAVELIN Lung 200): An open-label, randomised, phase 3 study. Lancet Oncol..

[119] Rittmeyer A., Barlesi F., Waterkamp D., Park K., Ciardiello F., von Pawel J., Gadgeel S.M., Hida T., Kowalski D.M., Dols M.C. (2017). Atezolizumab versus docetaxel in patients with previously treated non-small-cell lung cancer (OAK): A phase 3, open-label, multicentre randomised controlled trial. Lancet.

[120] Fehrenbacher L., Spira A., Ballinger M., Kowanetz M., Vansteenkiste J., Mazieres J., Park K., Smith D., Artal-Cortes A., Lewanski C. (2016). Atezolizumab versus docetaxel for patients with previously treated non-small-cell lung cancer (POPLAR): A multicentre, open-label, phase 2 randomised controlled trial. Lancet.

[121] Peters S., Gettinger S., Johnson M.L., Jänne P.A., Garassino M.C., Christoph D., Toh C.K., Rizvi N.A., Chaft J.E., Carcereny Costa E. (2017). Phase II Trial of Atezolizumab As First-Line or Subsequent Therapy for Patients With Programmed Death-Ligand 1-Selected Advanced Non-Small-Cell Lung Cancer (BIRCH). J. Clin. Oncol..

[122] Spigel D.R., Chaft J.E., Gettinger S., Chao B.H., Dirix L., Schmid P., Chow L.Q.M., Hicks R.J., Leon L., Fredrickson J. (2018). FIR: Efficacy, Safety, and Biomarker Analysis of a Phase II Open-Label Study of Atezolizumab in PD-L1-Selected Patients With NSCLC. J. Thorac. Oncol..

[123] Balar A.V., Galsky M.D., Rosenberg J.E., Powles T., Petrylak D.P., Bellmunt J., Loriot Y., Necchi A., Hoffman-Censits J., Perez-Gracia J.L. (2017). Atezolizumab as first-line treatment in cisplatin-ineligible patients with locally advanced and metastatic urothelial carcinoma: A single-arm, multicentre, phase 2 trial. Lancet.

[124] Powles T., Durán I., van der Heijden M.S., Loriot Y., Vogelzang N.J., De Giorgi U., Oudard S., Retz M.M., Castellano D., Bamias A. (2018). Atezolizumab versus chemotherapy in patients with platinum-treated locally advanced or metastatic urothelial carcinoma (IMvigor211): A multicentre, open-label, phase 3 randomised controlled trial. Lancet.

[125] Antonia S.J., Villegas A., Daniel D., Vicente D., Murakami S., Hui R., Yokoi T., Chiappori A., Lee K.H., de Wit M. (2017). Durvalumab after Chemoradiotherapy in Stage III Non-Small-Cell Lung Cancer. N. Engl. J. Med..

[126] Garassino M.C., Cho B.-C., Kim J.-H., Mazières J., Vansteenkiste J., Lena H., Corral Jaime J., Gray J.E., Powderly J., Chouaid C. (2018). Durvalumab as third-line or later treatment for advanced non-small-cell lung cancer (ATLANTIC): An open-label, single-arm, phase 2 study. Lancet Oncol..

[127] Qin S., Xu L., Yi M., Yu S., Wu K., Luo S. (2019). Novel immune checkpoint targets: Moving beyond PD-1 and CTLA-4. Mol. Cancer.

[128] Picardo S.L., Doi J., Hansen A.R. (2019). Structure and Optimization of Checkpoint Inhibitors. Cancers.

[129] Sharma A., Subudhi S.K., Blando J., Scutti J., Vence L., Wargo J., Allison J.P., Ribas A., Sharma P. (2019). Anti-CTLA-4 Immunotherapy Does Not Deplete FOXP3+ Regulatory T Cells (Tregs) in Human Cancers. Clin. Cancer Res..

[130] Fares C.M., Van Allen E.M., Drake C.G., Allison J.P., Hu-Lieskovan S. (2019). Mechanisms of Resistance to Immune Checkpoint Blockade: Why Does Checkpoint Inhibitor Immunotherapy Not Work for All Patients?. Am. Soc. Clin. Oncol. Educ. Book.

[131] Postow M.A., Chesney J., Pavlick A.C., Robert C., Grossmann K., McDermott D., Linette G.P., Meyer N., Giguere J.K., Agarwala S.S. (2015). Nivolumab and Ipilimumab versus Ipilimumab in Untreated Melanoma. N. Engl. J. Med..

[132] Hodi F.S., Chesney J., Pavlick A.C., Robert C., Grossmann K.F., McDermott D.F., Linette G.P., Meyer N., Giguere J.K., Agarwala S.S. (2016). Combined nivolumab and ipilimumab versus ipilimumab alone in patients with advanced melanoma: 2-year overall survival outcomes in a multicentre, randomised, controlled, phase 2 trial. Lancet Oncol..

[133] Larkin J., Chiarion-Sileni V., Gonzalez R., Grob J.J., Cowey C.L., Lao C.D., Schadendorf D., Dummer R., Smylie M., Rutkowski P. (2015). Combined Nivolumab and Ipilimumab or Monotherapy in Previously Untreated Melanoma. N. Engl. J. Med..

[134] Larkin J., Chiarion-Sileni V., Gonzalez R., Grob J.-J., Rutkowski P., Lao C.D., Cowey C.L., Schadendorf D., Wagstaff J., Dummer R. (2019). Five-Year Survival with Combined Nivolumab and Ipilimumab in Advanced Melanoma. N. Engl. J. Med..

[135] Motzer R.J., Tannir N.M., McDermott D.F., Arén Frontera O., Melichar B., Choueiri T.K., Plimack E.R., Barthélémy P., Porta C., George S. (2018). Nivolumab plus Ipilimumab versus Sunitinib in Advanced Renal-Cell Carcinoma. N. Engl. J. Med..

[136] Motzer R.J., Rini B.I., McDermott D.F., Frontera O.A., Hammers H.J., Carducci M.A., Salman P., Escudier B., Beuselinck B., Amin A. (2019). Nivolumab plus ipilimumab versus sunitinib in first-line treatment for advanced renal cell carcinoma: Extended follow-up of efficacy and safety results from a randomised, controlled, phase 3 trial. Lancet Oncol..

[137] Overman M.J., McDermott R., Leach J.L., Lonardi S., Lenz H.-J., Morse M.A., Desai J., Hill A., Axelson M., Moss R.A. (2017). Nivolumab in patients with metastatic DNA mismatch repair-deficient or microsatellite instability-high colorectal cancer (CheckMate 142): An open-label, multicentre, phase 2 study. Lancet Oncol..

[138] Overman M.J., Lonardi S., Wong K.Y.M., Lenz H.-J., Gelsomino F., Aglietta M., Morse M.A., Van Cutsem E., McDermott R., Hill A. (2018). Durable Clinical Benefit With Nivolumab Plus Ipilimumab in DNA Mismatch Repair-Deficient/Microsatellite Instability-High Metastatic Colorectal Cancer. J. Clin. Oncol..

[139] Janjigian Y.Y., Bendell J., Calvo E., Kim J.W., Ascierto P.A., Sharma P., Ott P.A., Peltola K., Jaeger D., Evans J. (2018). CheckMate-032 Study: Efficacy and Safety of Nivolumab and Nivolumab Plus Ipilimumab in Patients With Metastatic Esophagogastric Cancer. J. Clin. Oncol..

[140] D’Angelo S.P., Mahoney M.R., Van Tine B.A., Atkins J., Milhem M.M., Jahagirdar B.N., Antonescu C.R., Horvath E., Tap W.D., Schwartz G.K. (2018). Nivolumab with or without ipilimumab treatment for metastatic sarcoma (Alliance A091401): Two open-label, non-comparative, randomised, phase 2 trials. Lancet Oncol..

[141] Hellmann M.D., Ciuleanu T.-E., Pluzanski A., Lee J.S., Otterson G.A., Audigier-Valette C., Minenza E., Linardou H., Burgers S., Salman P. (2018). Nivolumab plus Ipilimumab in Lung Cancer with a High Tumor Mutational Burden. N. Engl. J. Med..

[142] Hellmann M.D., Paz-Ares L., Bernabe Caro R., Zurawski B., Kim S.-W., Carcereny Costa E., Park K., Alexandru A., Lupinacci L., de la Mora Jimenez E. (2019). Nivolumab plus Ipilimumab in Advanced Non–Small-Cell Lung Cancer. N. Engl. J. Med..

[143] Ready N.E., Ott P.A., Hellmann M.D., Zugazagoitia J., Hann C.L., de Braud F., Antonia S.J., Ascierto P.A., Moreno V., Atmaca A. (2020). Nivolumab Monotherapy and Nivolumab Plus Ipilimumab in Recurrent Small Cell Lung Cancer: Results From the CheckMate 032 Randomized Cohort. J. Thorac. Oncol..

[144] Antonia S.J., López-Martin J.A., Bendell J., Ott P.A., Taylor M., Eder J.P., Jäger D., Pietanza M.C., Le D.T., de Braud F. (2016). Nivolumab alone and nivolumab plus ipilimumab in recurrent small-cell lung cancer (CheckMate 032): A multicentre, open-label, phase 1/2 trial. Lancet Oncol..

[145] Scherpereel A., Mazieres J., Greillier L., Lantuejoul S., Dô P., Bylicki O., Monnet I., Corre R., Audigier-Valette C., Locatelli-Sanchez M. (2019). Nivolumab or nivolumab plus ipilimumab in patients with relapsed malignant pleural mesothelioma (IFCT-1501 MAPS2): A multicentre, open-label, randomised, non-comparative, phase 2 trial. Lancet Oncol..

[146] Syed Y.Y. (2017). Durvalumab: First global approval. Drugs.

[147] Siu L.L., Even C., Mesía R., Remenar E., Daste A., Delord J.-P., Krauss J., Saba N.F., Nabell L., Ready N.E. (2019). Safety and Efficacy of Durvalumab With or Without Tremelimumab in Patients With PD-L1–Low/Negative Recurrent or Metastatic HNSCC: The Phase 2 CONDOR Randomized Clinical Trial. JAMA Oncol..

[148] Ferris R.L., Haddad R., Even C., Tahara M., Dvorkin M., Ciuleanu T.E., Clement P.M., Mesia R., Kutukova S., Zholudeva L. Durvalumab with or without Tremelimumab in Patients with Recurrent or Metastatic Head and Neck Squamous Cell Carcinoma: EAGLE, a Randomized, Open-Label Phase III Study. https://www.sciencedirect.com/science/article/pii/S0923753420364346.

[149] O’Reilly E.M., Oh D.-Y., Dhani N., Renouf D.J., Lee M.A., Sun W., Fisher G.A., Hezel A.F., Chang S.-C., Vlahovic G. (2018). A randomized phase 2 study of durvalumab monotherapy and in combination with tremelimumab in patients with metastatic pancreatic ductal adenocarcinoma (mPDAC): ALPS study. JCO.

[150] Cho B.C., Lee K.H., Ahn M.-J., Geater S.L., Ngoc T.V., Wang C.-C., Cho E.K., Lee J.S., Sriuranpong V., Bui Q. (2019). 474O—Efficacy and safety of first-line durvalumab (D) ± tremelimumab (T) vs chemotherapy (CT) in Asian patients with metastatic NSCLC: Results from MYSTIC. Ann. Oncol..

[151] Andrews L.P., Yano H., Vignali D.A.A. (2019). Inhibitory receptors and ligands beyond PD-1, PD-L1 and CTLA-4: Breakthroughs or backups. Nat. Immunol..

[152] Tundo G.R., Sbardella D., Lacal P.M., Graziani G., Marini S. (2019). On the Horizon: Targeting Next-Generation Immune Checkpoints for Cancer Treatment. Chemotherapy.

[153] Koyama S., Akbay E.A., Li Y.Y., Herter-Sprie G.S., Buczkowski K.A., Richards W.G., Gandhi L., Redig A.J., Rodig S.J., Asahina H. (2016). Adaptive resistance to therapeutic PD-1 blockade is associated with upregulation of alternative immune checkpoints. Nat. Commun..

[154] Harjunpää H., Guillerey C. (2020). TIGIT as an emerging immune checkpoint. Clin. Exp. Immunol..

[155] Sanchez-Correa B., Valhondo I., Hassouneh F., Lopez-Sejas N., Pera A., Bergua J.M., Arcos M.J., Bañas H., Casas-Avilés I., Durán E. (2019). DNAM-1 and the TIGIT/PVRIG/TACTILE Axis: Novel Immune Checkpoints for Natural Killer Cell-Based Cancer Immunotherapy. Cancers.

[156] Whelan S., Ophir E., Kotturi M.F., Levy O., Ganguly S., Leung L., Vaknin I., Kumar S., Dassa L., Hansen K. (2019). PVRIG and PVRL2 Are Induced in Cancer and Inhibit CD8+ T-cell Function. Cancer Immunol. Res..

[157] André P., Denis C., Soulas C., Bourbon-Caillet C., Lopez J., Arnoux T., Bléry M., Bonnafous C., Gauthier L., Morel A. (2018). Anti-NKG2A mAb Is a Checkpoint Inhibitor that Promotes Anti-tumor Immunity by Unleashing Both T and NK Cells. Cell.

[158] Mingari M.C., Pietra G., Moretta L. (2019). Immune Checkpoint Inhibitors: Anti-NKG2A Antibodies on Board. Trends Immunol..

[159] Leone R.D., Emens L.A. (2018). Targeting adenosine for cancer immunotherapy. J. Immunother. Cancer.

[160] Hammami A., Allard D., Allard B., Stagg J. (2019). Targeting the adenosine pathway for cancer immunotherapy. Semin. Immunol..

[161] Folkes A.S., Feng M., Zain J.M., Abdulla F., Rosen S.T., Querfeld C. (2018). Targeting CD47 as a cancer therapeutic strategy: The cutaneous T-cell lymphoma experience. Curr. Opin. Oncol..

[162] Russ A., Hua A.B., Montfort W.R., Rahman B., Riaz I.B., Khalid M.U., Carew J.S., Nawrocki S.T., Persky D., Anwer F. (2018). Blocking “don’t eat me” signal of CD47-SIRPα in hematological malignancies, an in-depth review. Blood Rev..

[163] Tsao L.-C., Crosby E.J., Trotter T.N., Agarwal P., Hwang B.-J., Acharya C., Shuptrine C.W., Wang T., Wei J., Yang X. (2019). CD47 blockade augmentation of trastuzumab antitumor efficacy dependent on antibody-dependent cellular phagocytosis. JCI Insight.

[164] Treffers L.W., ten Broeke T., Rösner T., Jansen J.H.M., van Houdt M., Kahle S., Schornagel K., Verkuijlen P.J.J.H., Prins J.M., Franke K. (2020). IgA-Mediated Killing of Tumor Cells by Neutrophils Is Enhanced by CD47–SIRPα Checkpoint Inhibition. Cancer Immunol. Res..

[165] Weinmann S.C., Pisetsky D.S. (2019). Mechanisms of immune-related adverse events during the treatment of cancer with immune checkpoint inhibitors. Rheumatology.

[166] Han X., Vesely M.D. (2019). Stimulating T Cells Against Cancer With Agonist Immunostimulatory Monoclonal Antibodies. Int. Rev. Cell. Mol. Biol..

[167] Chu D.-T., Bac N.D., Nguyen K.-H., Tien N.L.B., Thanh V.V., Nga V.T., Ngoc V.T.N., Anh Dao D.T., Hoan L.N., Hung N.P. (2019). An Update on Anti-CD137 Antibodies in Immunotherapies for Cancer. Int. J. Mol. Sci..

[168] Polesso F., Sarker M., Weinberg A.D., Murray S.E., Moran A.E. (2019). OX40 Agonist Tumor Immunotherapy Does Not Impact Regulatory T Cell Suppressive Function. J. Immunol..

[169] Kvarnhammar A.M., Veitonmäki N., Hägerbrand K., Dahlman A., Smith K.E., Fritzell S., von Schantz L., Thagesson M., Werchau D., Smedenfors K. (2019). The CTLA-4 x OX40 bispecific antibody ATOR-1015 induces anti-tumor effects through tumor-directed immune activation. J. Immunother. Cancer.

[170] Gutiérrez A., Rodríguez J., Martínez J., Amezaga R., Ramos R., Galmes B., Bea M.D., Ferrer J., Pons J., Sampol A. (2006). Pathogenic study of anti-CD20 infusion-related severe refractory shock in diffuse large B-cell lymphoma. Leuk. Lymphoma.

[171] Facciabene A., De Sanctis F., Pierini S., Reis E.S., Balint K., Facciponte J., Rueter J., Kagabu M., Magotti P., Lanitis E. (2017). Local endothelial complement activation reverses endothelial quiescence, enabling t-cell homing, and tumor control during t-cell immunotherapy. Oncoimmunology.

[172] Hb J., Rm S., Argiris A., Je B., Lp K., Rl F. (2017). Increased PD-1+ and TIM-3+ TILs during Cetuximab Therapy Inversely Correlate with Response in Head and Neck Cancer Patients. Cancer Immunol. Res..

[173] Chaganty B.K.R., Qiu S., Gest A., Lu Y., Ivan C., Calin G.A., Weiner L.M., Fan Z. (2018). Trastuzumab upregulates PD-L1 as a potential mechanism of trastuzumab resistance through engagement of immune effector cells and stimulation of IFNγ secretion. Cancer Lett..

[174] Okita R., Maeda A., Shimizu K., Nojima Y., Saisho S., Nakata M. (2017). PD-L1 overexpression is partially regulated by EGFR/HER2 signaling and associated with poor prognosis in patients with non-small-cell lung cancer. Cancer Immunol. Immunother..

[175] Zhang N., Zeng Y., Du W., Zhu J., Shen D., Liu Z., Huang J.-A. (2016). The EGFR pathway is involved in the regulation of PD-L1 expression via the IL-6/JAK/STAT3 signaling pathway in EGFR-mutated non-small cell lung cancer. Int. J. Oncol..

[176] Bylicki O., Paleiron N., Margery J., Guisier F., Vergnenegre A., Robinet G., Auliac J.-B., Gervais R., Chouaid C. (2017). Targeting the PD-1/PD-L1 Immune Checkpoint in EGFR-Mutated or ALK-Translocated Non-Small-Cell Lung Cancer. Target. Oncol..

[177] Taberna M., Oliva M., Mesía R. (2019). Cetuximab-Containing Combinations in Locally Advanced and Recurrent or Metastatic Head and Neck Squamous Cell Carcinoma. Front. Oncol..

[178] Cioroianu A.I., Stinga P.I., Sticlaru L., Cioplea M.D., Nichita L., Popp C., Staniceanu F. (2019). Tumor Microenvironment in Diffuse Large B-Cell Lymphoma: Role and Prognosis. Anal. Cell. Pathol. (Amst.).

[179] Xie M., Huang X., Ye X., Qian W. (2019). Prognostic and clinicopathological significance of PD-1/PD-L1 expression in the tumor microenvironment and neoplastic cells for lymphoma. Int. Immunopharmacol..

[180] Timmerman J., Herbaux C., Ribrag V., Zelenetz A.D., Houot R., Neelapu S.S., Logan T., Lossos I.S., Urba W., Salles G. (2020). Urelumab alone or in combination with rituximab in patients with relapsed or refractory B-cell lymphoma. Am. J. Hematol..

[181] Zhao J., Song Y., Liu D. (2019). Recent advances on blinatumomab for acute lymphoblastic leukemia. Exp. Hematol. Oncol..

[182] Gökbuget N. (2020). Clinical Experience with Bispecific T Cell Engagers. Recent Results Cancer Res..

[183] Van Dam P.A., Verhoeven Y., Trinh X.B., Wouters A., Lardon F., Prenen H., Smits E., Baldewijns M., Lammens M. (2019). RANK/RANKL signaling inhibition may improve the effectiveness of checkpoint blockade in cancer treatment. Crit. Rev. Oncol. Hematol..

[184] Gonzalez-Suarez E., Jacob A.P., Jones J., Miller R., Roudier-Meyer M.P., Erwert R., Pinkas J., Branstetter D., Dougall W.C. (2010). RANK ligand mediates progestin-induced mammary epithelial proliferation and carcinogenesis. Nature.

[185] Palafox M., Ferrer I., Pellegrini P., Vila S., Hernandez-Ortega S., Urruticoechea A., Climent F., Soler M.T., Muñoz P., Viñals F. (2012). RANK induces epithelial-mesenchymal transition and stemness in human mammary epithelial cells and promotes tumorigenesis and metastasis. Cancer Res..

[186] Smyth E.C., Fassan M., Cunningham D., Allum W.H., Okines A.F.C., Lampis A., Hahne J.C., Rugge M., Peckitt C., Nankivell M. (2016). Effect of Pathologic Tumor Response and Nodal Status on Survival in the Medical Research Council Adjuvant Gastric Infusional Chemotherapy Trial. J. Clin. Oncol..

[187] Afzal M.Z., Shirai K. (2018). Immune checkpoint inhibitor (anti-CTLA-4, anti-PD-1) therapy alone versus immune checkpoint inhibitor (anti-CTLA-4, anti-PD-1) therapy in combination with anti-RANKL denosumuab in malignant melanoma: A retrospective analysis at a tertiary care center. Melanoma Res..

[188] Ahern E., Harjunpää H., Barkauskas D., Allen S., Takeda K., Yagita H., Wyld D., Dougall W.C., Teng M.W.L., Smyth M.J. (2017). Co-administration of RANKL and CTLA4 Antibodies Enhances Lymphocyte-Mediated Antitumor Immunity in Mice. Clin. Cancer Res..

[189] Haibe Y., Kreidieh M., El Hajj H., Khalifeh I., Mukherji D., Temraz S., Shamseddine A. (2020). Resistance Mechanisms to Anti-angiogenic Therapies in Cancer. Front. Oncol..

[190] Ricklin D., Hajishengallis G., Yang K., Lambris J.D. (2010). Complement: A key system for immune surveillance and homeostasis. Nat. Immunol..

[191] Reis E.S., Mastellos D.C., Hajishengallis G., Lambris J.D. (2019). New insights into the immune functions of complement. Nat. Rev. Immunol..

[192] Pio R., Ajona D., Ortiz-Espinosa S., Mantovani A., Lambris J.D. (2019). Complementing the Cancer-Immunity Cycle. Front Immunol.

[193] Ajona D., Ortiz-Espinosa S., Pio R., Lecanda F. (2019). Complement in Metastasis: A Comp in the Camp. Front. Immunol..

[194] Ajona D., Ortiz-Espinosa S., Moreno H., Lozano T., Pajares M.J., Agorreta J., Bértolo C., Lasarte J.J., Vicent S., Hoehlig K. (2017). A Combined PD-1/C5a Blockade Synergistically Protects against Lung Cancer Growth and Metastasis. Cancer Discov..

[195] Zha H., Han X., Zhu Y., Yang F., Li Y., Li Q., Guo B., Zhu B. (2017). Blocking C5aR signaling promotes the anti-tumor efficacy of PD-1/PD-L1 blockade. OncoImmunology.

[196] Lind H., Gameiro S.R., Jochems C., Donahue R.N., Strauss J., MD Gulley J.L., Palena C., Schlom J. (2020). Dual targeting of TGF-β and PD-L1 via a bifunctional anti-PD-L1/TGF-βRII agent: Status of preclinical and clinical advances. J. Immunother. Cancer.

[197] Dougall W.C., Roman Aguilera A., Smyth M.J. (2019). Dual targeting of RANKL and PD-1 with a bispecific antibody improves anti-tumor immunity. Clin. Transl. Immunol..

[198] Herrera-Camacho I., Anaya-Ruiz M., Perez-Santos M., Millán-Pérez Peña L., Bandala C., Landeta G. (2019). Cancer immunotherapy using anti-TIM3/PD-1 bispecific antibody: A patent evaluation of EP3356411A1. Expert Opin. Ther. Pat..

[199] Cohen E.E.W., Pishvaian M.J., Shepard D.R., Wang D., Weiss J., Johnson M.L., Chung C.H., Chen Y., Huang B., Davis C.B. (2019). A phase Ib study of utomilumab (PF-05082566) in combination with mogamulizumab in patients with advanced solid tumors. J Immunother. Cancer.

[200] Claus C., Ferrara C., Xu W., Sam J., Lang S., Uhlenbrock F., Albrecht R., Herter S., Schlenker R., Hüsser T. (2019). Tumor-targeted 4-1BB agonists for combination with T cell bispecific antibodies as off-the-shelf therapy. Sci. Transl. Med..

[201] Koopmans I., Hendriks D., Samplonius D.F., van Ginkel R.J., Heskamp S., Wierstra P.J., Bremer E., Helfrich W. (2018). A novel bispecific antibody for EGFR-directed blockade of the PD-1/PD-L1 immune checkpoint. Oncoimmunology.

[202] Buatois V., Johnson Z., Salgado-Pires S., Papaioannou A., Hatterer E., Chauchet X., Richard F., Barba L., Daubeuf B., Cons L. (2018). Preclinical Development of a Bispecific Antibody that Safely and Effectively Targets CD19 and CD47 for the Treatment of B-Cell Lymphoma and Leukemia. Mol. Cancer Ther..

[203] Golay J., Regazzi M. (2020). Key Features Defining the Disposition of Bispecific Antibodies and Their Efficacy In Vivo. Ther. Drug Monit..

[204] Golay J., Choblet S., Iwaszkiewicz J., Cérutti P., Ozil A., Loisel S., Pugnière M., Ubiali G., Zoete V., Michielin O. (2016). Design and Validation of a Novel Generic Platform for the Production of Tetravalent IgG1-like Bispecific Antibodies. J. Immunol..

[205] Klein C., Schaefer W., Regula J.T. (2016). The use of CrossMAb technology for the generation of bi- and multispecific antibodies. MAbs.

[206] Davda J., Declerck P., Hu-Lieskovan S., Hickling T.P., Jacobs I.A., Chou J., Salek-Ardakani S., Kraynov E. (2019). Immunogenicity of immunomodulatory, antibody-based, oncology therapeutics. J. Immunother. Cancer.

