# Supplementary material for: Combined Anti-Cancer Strategies Based on Anti-Checkpoint Inhibitor Antibodies
Source: Antibodies (Basel). 2020 May 20;9(2):17. doi: 10.3390/antib9020017 (PMC7345008; doi:10.3390/antib9020017)
Supplement: Supplementary file 1 [file antibodies-09-00017-s001.pdf]

Supplementary Table 1. Results of major Phase II and III clinical trials using anti-ICI as monotherapy

Target Antigen	Drug	Cancer type	Phase<br>NCT number	Study Population	Outcome	Source
CTLA-4	Tremelimumab	Advanced Melanoma	Phase III<br>NCT00257205	655	***Tremelimumab 15 mg/kg q90d***<br>ORR: 10.7%<br>OS: 12.6 mo<br>DOR: 35.8 mo<br>***Chemotherapy***<br>ORR: 9.8%<br>OS. 10.7 mo<br>DOR: 13.7 mo	[68]
		Pleural or Peritoneal Malignant Mesothelioma	Phase II<br>NCT01843374	571	***Tremelimumab 10 mg/kg q4w***<br>ORR: 4.5%<br>OS:7.7 mo<br>PFS: 2.7 mo<br>***Placebo***<br>ORR: 1.1%<br>OS:7.3 mo<br>PFS: 2.8 mo	[69]
CTLA-4	Ipilimumab	Stage III-IV Melanoma	Phase III<br>NCT00094653	676	***Ipilimumab 3mg/kg q3w***<br>OS: 10.1 mo<br>***Ipilimumab 3mg/kg q3w* + gp100 vaccine**<br>OS: 10.0 mo<br>**gp100 vaccine**<br>OS: 6.4 mo	[47]
		Stage III-IV Melanoma	Phase II<br>NCT01696045	14	***Ipilimumab 3mg/kg q3w***<br>ORR: 0.0%<br>OS at 1 yr: 75%<br>PFS: 2.6 mo<br>***Ipilimumab 10mg/kg q3w***<br>*ORR: 25.0%*<br>OS at 1 year: 62.5%<br>PFS: 2.9 mo	[72]

CTLA-4	Ipililumab	Stage III Post Resection Melanoma	Phase III<br>NCT00636168	951	***Ipilimumab 10mg/kg q3w***<br>OS at 1 yr: 93.5%<br>OS at 5 yrs: 65.4%<br>RFS: 26.1 mo<br>***Placebo***<br>OS at 1 yr: 87.3%<br>OS at 5 yrs: 54.4%<br>RFS: 17.0 mo	[73]
		Metastatic Melanoma	Phase III<br>NCT01515189	727	***Ipilimumab 3mg/kg q3w***<br>ORR: 12.2%<br>OS*:* 11.5 mo<br>PFS: 2.8 mo<br>***Ipilimumab 10mg/kg q3w***<br>*ORR:* 15.3%<br>OS*:* 15.7 mo<br>PFS: *2.8 mo*	[74]
		Advanced Melanoma	Phase III<br>NCT01866319	834	***Ipilimumab 3mg/kg q3w***<br>ORR:3.0%<br>OS: 15.9 mo<br>PFS: 3.4 mo<br>***Pembrolizumab 10mg/kg q2w/q3w***<br>ORR: 14.0%<br>OS: 32.7 mo<br>PFS: 8.7 mo	[75]
		Gastric and Gastroesophageal Junction Cancer	Phase II<br>NCT01585987	143	***Ipilimumab 10 mg/kg q4w***<br>OS: 16.8 mo<br>PFS: 2.7 mo<br>***Chemotherapy***<br>OS: 12.1 mo<br>PFS:4.9 mo	[76]
		Recurrent Ovarian Cancer	Phase II<br>NCT01611558	49	***Ipilimumab 10mg/kg q3w***<br>ORR: 10.3%	[77]
		Metastatic Prostate Cancer	Phase II<br>NCT02279862	82	***Ipilimumab 3mg/kg q3w***<br>OS at 1 yr*:* 15.4%<br>rPFS at 1yr*:* 15.4%<br>***Ipilimumab 10mg/kg q3w***<br>OS at 1yr*:* 22.2%<br>rPFS at 1 yr*:* 11.1%	www.<br>clinicaltrials.gov

CTLA-4	Ipililumab	Advanced Prostate Cancer	Phase III NCT01057810	837	***Ipilimumab 10mg/kg q3w***<br>OS*:* 28.6 mo<br>PFS*:* 5.6 mo<br>***Placebo****:*<br>*OS:* 29.7 mo<br>PFS*:* 3.8 mo	www. clinicaltrials.gov
PD-1	Nivolumab	Advanced (anti-CTLA-4 resistant) Melanoma	Phase III NCT01721746	631	***Nivolumab 3 mg/kg q2w***<br>ORR*:* 27.2%<br>OS*:* 15.7 mo<br>PFS*:* 3.1 mo<br>***Chemotherapy***<br>ORR: 9.8%<br>OS: 14.4 mo<br>PFS: 3.6 mo	[78]
		Untreated Advanced Melanoma	Phase III NCT01721772	418	***Nivolumab 3 mg/kg q2w***<br>ORR*:* 42.9%<br>OS*:* 37.5 mo<br>PFS*:* 5.1 mo<br>***Dacarbazine***<br>*ORR:*14.4%<br>*OS:* 11.2 mo<br>*PFS:* 2.2 mo	[79]
		Metastatic or Unresectable Bladder Cancer	Phase II NCT02387996	270	***Nivolumab 3 mg/kg q2w***<br>ORR: 19.6%<br>OS: 8.7 mo<br>PFS: 2.0 mo	[84]
		Recurrent or Metastatic Head and Neck Squamous Cell Carcinoma	Phase III NCT02105636	361	***Nivolumab 3 mg/kg q2w***<br>ORR: 13.3%<br>OS*:* 7.5 mo<br>PFS : 2.0 mo<br>***Cetuximab/methotrexate/docetaxel***<br>ORR: 5.8%<br>OS: 5.1 mo<br>PFS : 2.3 mo	[85]
		Recurrent/metastatic Nasopharyngeal Cancer	Phase II NCT02339558	45	***Nivolumab 3 mg/kg q2w***<br>ORR: 20.5%<br>OS: 17.1 mo<br>PFS: 2.8 mo	[86]

PD-1	Nivolumab	Advanced/Metastatic NSCLC	Phase II<br>NCT01721759	117	***Nivolumab 3 mg/kg q2w***<br>**ORR:** 14.5%<br>**OS:** 8.2 mo<br>**PFS :** 1.9 mo	[87]
		Advanced/Metastatic NSCLC	Phase III<br>NCT02613507	639	***Nivolumab 3 mg/kg q2w***<br>ORR : 16.6%<br>OS*:* 12.0 mo<br>PFS*: 2.8* mo<br>***Docetaxel***<br>*ORR : 4.2%*<br>OS*:* 9.6 mo<br>PFS*:* 2.8 mo	[88]
		Advanced/Metastatic NSCLC	Phase III<br>NCT01642004	352	***Nivolumab 3 mg/kg q2w***<br>ORR*:* 20.0%<br>OS*:* 9.2 mo<br>PFS *: 3.5* mo<br>***Docetaxel***<br>*ORR: 8.8%*<br>OS*:* 6.0 mo<br>PFS*:* 2.8 mo	[89]
		Metastatic NSCLC	Phase III<br>NCT01673867	582	***Nivolumab 3 mg/kg q2w***<br>ORR*:* 19.2%<br>OS*:* 12.2 mo<br>PFS*: 2.3* mo<br>***Docetaxel***<br>*ORR:* 12.4%<br>OS*:* 9.4 mo<br>PFS*:* 4.2 mo	[90]
		Stage IV or Recurrent NSCLC not previously treated with systemic therapy	Phase III<br>NCT02041533	541	***Nivolumab 3 mg/kg q2w***<br>ORR*:* 26%<br>OS*:* 14.4 mo<br>PFS*:* 4.2 mo<br>***Chemotherapy***<br>*ORR: 33%*<br>OS*:* 13.2 mo<br>PFS*:* 5.9 mo	[91]

PD-1	Nivolumab	Advanced/Metastatic Renal Cell Carcinoma	Phase III NCT01668784	822	***Nivolumab 3 mg/kg q2w***<br>ORR*:* 25.1%<br>OS*:* 25.0 mo<br>***Everolimus***<br>*ORR:* 5.4%<br>OS*:* 19.6 mo	[92]
		Advanced/Metastatic Renal Cell Carcinoma	Phase II NCT01354431	168	***Nivolumab 0.3, 2, 10 mg/kg q3w***<br>**ORR :**<br>*0.3mg/kg:* 20.0%<br>*2.0mg/kg:* 22.2%<br>*10mg/kg :* 20.4%<br>**OS :**<br>*0.3mg/kg:* 10.4 mo<br>*2.0mg/kg:* 25.5 mo<br>*10mg/kg :* 24.8 mo<br>**PFS :**<br>*0.3mg/kg:* 2.7 mo<br>*2.0mg/kg:* 4.0 mo<br>*10mg/kg:* 4.2 mo	[93]
		R/R Follicular Lymphoma	Phase II NCT02038946	92	***Nivolumab 3 mg/kg q2w***<br>ORR : 4.3%<br>PFS : 2.2 mo	[80]
		R/R DLBCL	Phase II NCT02038933	121	***Nivolumab 3 mg/kg q2w***<br>***Autologous stem cell transplant failed [N=87]***<br>ORR: 10.3%<br>OS: 12.2 mo<br>PFS: 1.9 mo<br>***Autologous stem cell transplant ineligible [N=34]***<br>ORR: 2.9%<br>OS: 5.8 mo<br>PFS1.4 mo	[81]
		R/R Classical HL	Phase II NCT02181738	243	***Nivolumab 3 mg/kg q2w***<br>ORR: 69%<br>OS: NR<br>PFS: 14.7 mo	[82,83]

PD-1	Pembrolizumab	High risk Melanoma	Phase III NCT02362594	1019	***Pembrolizumab 200 mg q3w***<br>1-year RFS*:* 75.4%<br>1.5-year RFS: 71.4<br>***Placebo***<br>1-yr RFS: 61.0%<br>1.5-year RFS: 53.2%	[97]
		Advanced/ metastatic Esophageal Carcinoma	Phase II NCT02559687	121	***Pembrolizumab 200 mg q3w***<br>ORR: 9.9%<br>OS: 5.8 mo<br>PFS: 2.0 mo	[99]
		Advanced Gastric and Gastroesophageal Junction Adenocarcinoma	Phase III NCT02370498	592	***Pembrolizumab 200 mg q3w***<br>ORR*:* 11.1%<br>OS*:* 9.1 mo<br>PFS*:* 1.5 mo<br>***Paclitaxel***<br>*ORR:* 12.5%<br>OS*:* 8.3 mo<br>PFS*:* 4.1 mo	[100]
		Recurrent Head and Neck Squamous Cell Carcinoma	Phase II NCT02255097	172	***Pembrolizumab 200 mg q3w***<br>ORR: 16.4%<br>OS: 8.4 mo<br>PFS: 2.1 mo	www. clinicaltrials.gov
		Recurrent Head and Neck Squamous Cell Carcinoma	Phase III NCT02252042	247	***Pembrolizumab 200 mg q3w***<br>ORR*:* 14.6%<br>OS*:* 8.4 mo<br>PFS*:* 2.1 mo<br>***Active Comparator***<br>*ORR:* 10.1%<br>OS*:* 6.9 mo<br>PFS*:* 2.3 mo	[101]

PD-1	Pembrolizumab	Relapsed NSCLC (Post-therapy)	Phase II/III NCT01905657	1034	***Pembrolizumab 2 mg/kg q3w***<br>ORR*:* 18.0%<br>*OS:* 10.4 mo<br>PFS: 3.9 mo<br>***Pembrolizumab 10 mg/kg q3w*:**<br>ORR: 18.5%<br>OS: 12.7 mo<br>PFS: 4.0 mo<br>***Docetaxel***<br>ORR: 9.3 %<br>OS: 8.5 mo<br>PFS: 4.0 mo	[102]
		Metastatic NSCLC	Phase III NCT02142738	305	***Pembrolizumab 200 mg q3w***<br>ORR*:* 44.8%<br>OS at 6 months*:* 80.2%<br>PFS*:* 10.3 mo<br>***Chemotherapy***<br>ORR: 27.8%<br>OS at 6 months*:* 72.4%<br>PFS*:* 6.0 mo	[103]
		PDL-1 positive advanced/ metastatic NSCLC	Phase III NCT02220894	1274	**Tumor with PD-L1+: ≥50% - ≥20% - ≥1%**<br>***Pembrolizumab 200 mg q3w***<br>*ORR:* 39.5% - 33.4% - 27.3%<br>OS*:* 20.0 mo - 17.7 mo - 16.7 mo<br>PFS: 7.1 mo - 6.2 mo - 5.4 mo<br>***Chemotherapy***<br>ORR: 32.0% - 28.9% - 26.5%<br>OS*:* 12.2 mo -13.0 mo - 12.1 mo<br>PFS: 6.4 mo - 6.6 mo - 6.5 mo	[104]
		Advanced Urothelial Cancer	Phase II NCT02335424	374	***Pembrolizumab 200 mg q3w***<br>ORR*:* 30.5%<br>OS*:*11.3 mo<br>PFS*:* 2.2 mo	[105]

			Phase III<br>NCT02256436	542	***Pembrolizumab 200 mg q3w***<br>ORR: 21.1%<br>1 yr OS: 44.2%<br>PFS: 2.1 mo<br>***Active Comparator***<br>ORR: 11.0%<br>1-year OS: 29.8%<br>PFS: 3.3 mo	[106]
PD-1	Pembrolizumab	R/R cHL	Phase II<br>NCT02453594	210	***Pembrolizumab 200 mg q3w***<br>ORR: 71.9%<br>OS 24 mo: 84.2 %<br>PFS: 13.7 mo	[107]
PD-1	Cemiplimab	Advanced Squamous cell carcinoma	Phase II<br>NCT02760498	78	***Cemiplimab 3mg/kg q2w***<br>ORR: 44.0%<br>1-year OS: 93.0%	[108]
PD-L1	Atezolizumab	Advanced/metastatic NSCLC	Phase III<br>[NCT02008227]	1225	***Atezolizumab 1200 mg q3w***<br>OS: 13.8 mo<br>PFS: 2.8 mo<br>***Docetaxel***<br>OS: 9.6 mo<br>PFS: 4.0 mo	[119]
		Naïve Locally Advanced (A), recurrent (B) and recurrent with brain metastatic NSCLC (C)	Phase II<br>NCT01846416	138<br>(A: 31,<br>B: 91, C:13)	***Atezolizumab 1200 mg q3w***<br>**ORR:**<br>A: 32%<br>B: 21%<br>C: 23%<br>**OS:**<br>A: 14.4 mo<br>B: 9.3 mo<br>*C:* 6.8 mo<br>**PFS:**<br>A: 4.7 mo<br>B: 2.5 mo<br>C: 2.7 mo	[122]
		Advanced/metastatic NSCLC ≥5% PD-L1+	Phase II<br>NCT02031458	659	***Atezolizumab 1200 mg q3w***<br>OS: 14.6 mo	[121]

PD-L1	Atezolizumab	Locally Advanced or Metastatic Recurrent NSCLC	Phase II NCT01903993	144	***Atezolizumab 1200 mg q3w***<br>ORR*:* 15.3%<br>OS*:* 12.6 mo<br>PFS*:* 2.7 mo<br>***Docetaxel***<br>*ORR:* 14.7%<br>OS*:* 9.7 mo<br>PFS*:* 3.4 mo	[120]
		Locally Advanced or Metastatic Urothelial Bladder Cancer	Phase II NCT02951767	119	***Atezolizumab 1200 mg q3w***<br>OS: 15.9 mo<br>PFS: 2.7 mo	www. clinicaltrials.gov
		Locally Advanced or Metastatic Urothelial Bladder Cancer	Phase II NCT02108652	123	***Atezolizumab 1200 mg q3w***<br>ORR: 23.0%<br>OS: 15.9 mo<br>PFS: 2.7 mo	[123]
		Locally Advanced or Metastatic Urothelial Bladder Cancer	Phase III NCT02302807	931	***Atezolizumab 1200 mg q3w***<br>ORR*:* 23.0%<br>OS*:* 11.1 mo<br>PFS*:* 2.1 mo<br>***Chemotherapy***<br>ORR: 22.0%<br>OS*:* 10.6 mo<br>PFS*:* 4.0 mo	[124]
PD-L1	Avelumab	Recurrent, Locally Advanced or metastatic Gastric & Gastro-oesophageal Junction Cancer	Phase III NCT02625623 (1st *endpoint not reached*)	371	***Avelumab 10 mg/kg q2w***<br>ORR: 2.2%<br>OS: 4.6 mo<br>PFS: 1.4 mo<br>***Chemotherapy***<br>*ORR:* 4.3%<br>OS*:* 5.0 mo<br>PFS*:* 2.7 mo	[115]
		Merkle Cell Carcinoma	Phase II NCT02155647	88	***Avelumab 10 mg/kg q2w***<br>ORR*:* 33.0%<br>OS*:* 12.9 mo<br>1-year PFS*:* 30%	[116,117]

PD-L1	Avelumab	NSCLC	Phase III<br>NCT02395172	792	***Avelumab 10 mg/kg q2w***<br>OS*:* 11.4 mo<br>PFS*:* 2.8 mo<br>***Docetaxel***<br>OS:10.3 mo<br>PFS*:* 4.2 mo	[118]
PD-L1	Durvalumab	Stage III NSCLC (Post-Chemoradiation Therapy)	Phase III<br>NCT02125461	713	***Durvalumab 10 mg/kg q2w***<br>ORR*:* 28.4%<br>PFS*:* 16.8 mo<br>***Placebo***<br>*ORR:*16.0 %<br>*PFS:* 5.6 mo	[125]
		Locally Advanced or Metastatic NSCLC	Phase II<br>NCT02087423	444	***Durvalumab 10 mg/kg q2w***<br>*Group 1 [N=28-30]: EGFR+/ALK+ PD-L1<25%*<br>ORR: 3.6%<br>OS: 9.9 mo<br>PFS: 1.9 mo<br>*Group 2 [N=74-77]: EGFR+/ALK+ PD-L1≥25%*<br>ORR: 12.1%<br>OS: 13.3 mo<br>PFS: 1.9 mo<br>*Group 3 [N=93-94]: EGFR-/ALK- PD-L1<25%*<br>ORR:7.5%<br>OS: 9.3 mo<br>PFS: 1.9 mo<br>*Group 4 [N=146-149]: EGFR+/ALK+ PD-L1≥25%*<br>ORR: 16.4%<br>OS: 10.9 mo<br>PFS: 3.3 mo<br>*Group 5 [N=67-69]: EGFR-/ALK- PD-L1≥90%*<br>ORR:30.9%<br>OS: NR<br>PFS: 2.4	[126]

DOR: Duration Of Response; ORR: Overall Response Rate; OS: Overall Survival; PFS: Progression-Free Survival; RFS: Relapse-Free Survival
